# Network medicine analysis of COPD multimorbidities

**DOI:** 10.1186/s12931-014-0111-4

**Published:** 2014-09-24

**Authors:** Solène Grosdidier, Antoni Ferrer, Rosa Faner, Janet Piñero, Josep Roca, Borja Cosío, Alvar Agustí, Joaquim Gea, Ferran Sanz, Laura I Furlong

**Affiliations:** Integrative Biomedical Informatics Group, Research Program on Biomedical Informatics (GRIB), IMIM (Hospital del Mar Research Institute), Barcelona, DCEXS, Universitat Pompeu Fabra, Barcelona, Spain; Servei de Pneumologia, IMIM (Hospital del Mar Research Institute), Barcelona, DCEXS, Universitat Pompeu Fabra, CIBERES, Barcelona, Spain; FISIB, CIBERES, Palma de Mallorca, Spain; Thorax Institute, Hospital Clínic, IDIBAPS, Universitat Barcelona, Barcelona, Spain; Hospital Universitari Son Espases-IdISPa, Ciberes, Palma de Mallorca, Spain

**Keywords:** Diseasome, Systems biology, Network medicine, Comorbidity, Multimorbidity, COPD, Tobacco chemicals

## Abstract

**Background:**

Patients with chronic obstructive pulmonary disease (COPD) often suffer concomitant disorders that worsen significantly their health status and vital prognosis. The pathogenic mechanisms underlying COPD multimorbidities are not completely understood, thus the exploration of potential molecular and biological linkages between COPD and their associated diseases is of great interest.

**Methods:**

We developed a novel, unbiased, integrative network medicine approach for the analysis of the diseasome, interactome, the biological pathways and tobacco smoke exposome, which has been applied to the study of 16 prevalent COPD multimorbidities identified by clinical experts.

**Results:**

Our analyses indicate that all COPD multimorbidities studied here are related at the molecular and biological level, sharing genes, proteins and biological pathways. By inspecting the connections of COPD with their associated diseases in more detail, we identified known biological pathways involved in COPD, such as inflammation, endothelial dysfunction or apoptosis, serving as a proof of concept of the methodology. More interestingly, we found previously overlooked biological pathways that might contribute to explain COPD multimorbidities, such as hemostasis in COPD multimorbidities other than cardiovascular disorders, and cell cycle pathway in the association of COPD with depression. Moreover, we also observed similarities between COPD multimorbidities at the pathway level, suggesting common biological mechanisms for different COPD multimorbidities. Finally, chemicals contained in the tobacco smoke target an average of 69% of the identified proteins participating in COPD multimorbidities.

**Conclusions:**

The network medicine approach presented here allowed the identification of plausible molecular links between COPD and comorbid diseases, and showed that many of them are targets of the tobacco exposome, proposing new areas of research for understanding the molecular underpinning of COPD multimorbidities.

**Electronic supplementary material:**

The online version of this article (doi:10.1186/s12931-014-0111-4) contains supplementary material, which is available to authorized users.

## Background

Multimorbidities, including cardiovascular diseases (CVD), skeletal muscle weakness, osteoporosis, metabolic syndrome, depression and lung cancer, among others, are highly prevalent in patients with chronic obstructive pulmonary disease (COPD) [1–4] and contribute to worsen their health-status and vital prognosis [4,5]. The pathogenic mechanisms linking COPD and its concomitant diseases are incompletely understood [6], but shared risk factors (tobacco smoking, physical inactivity, ageing) and COPD-related specific mechanisms (systemic inflammation, tissue hypoxia, abnormal protein metabolism) may potentially contribute. It is also possible that COPD and its multimorbidities share genes, proteins and pathways that explain their tendency to co-occur together in a particular patient [7–9]. A systematic investigation of this "*shared component hypothesis*" can provide insights to improve the prevention, early diagnosis, prognosis and/or treatment of COPD multimorbidities [9,10]. The new discipline of network medicine offers a platform to systematically explore the molecular complexity of a given disease with the potential to identify new molecular relationships among apparently distinct clinical manifestations [9,11].

In this study, we sought to test the *shared component hypothesis* in COPD. Under this hypothesis, the multimorbidities of COPD most frequently seen in the clinic would be related between them and with COPD at the molecular level by common genes, proteins and biological pathways. To this end, we used a network medicine approach that included: *(1)* data mining of the diseasome (or disease network), the interactome (as defined by a protein-protein interaction (PPI) network) to enrich this diseasome and the tobacco smoking exposome (representing the exposure to tobacco smoke chemicals, the main risk factor for COPD); and, *(2)* a functional analysis to identify the biological pathways potentially involved in COPD multimorbidity. Our findings indicate that the most prevalent COPD multimorbidities are likely to be related at the molecular level, and highlight some previously overlooked pathways that might contribute to explain their co-occurrence. Remarkably, the results here presented propose new hypothesis for explaining the molecular underpinning of COPD multimorbidities.

## Methods

### Disease vocabulary

**Figure 1:**
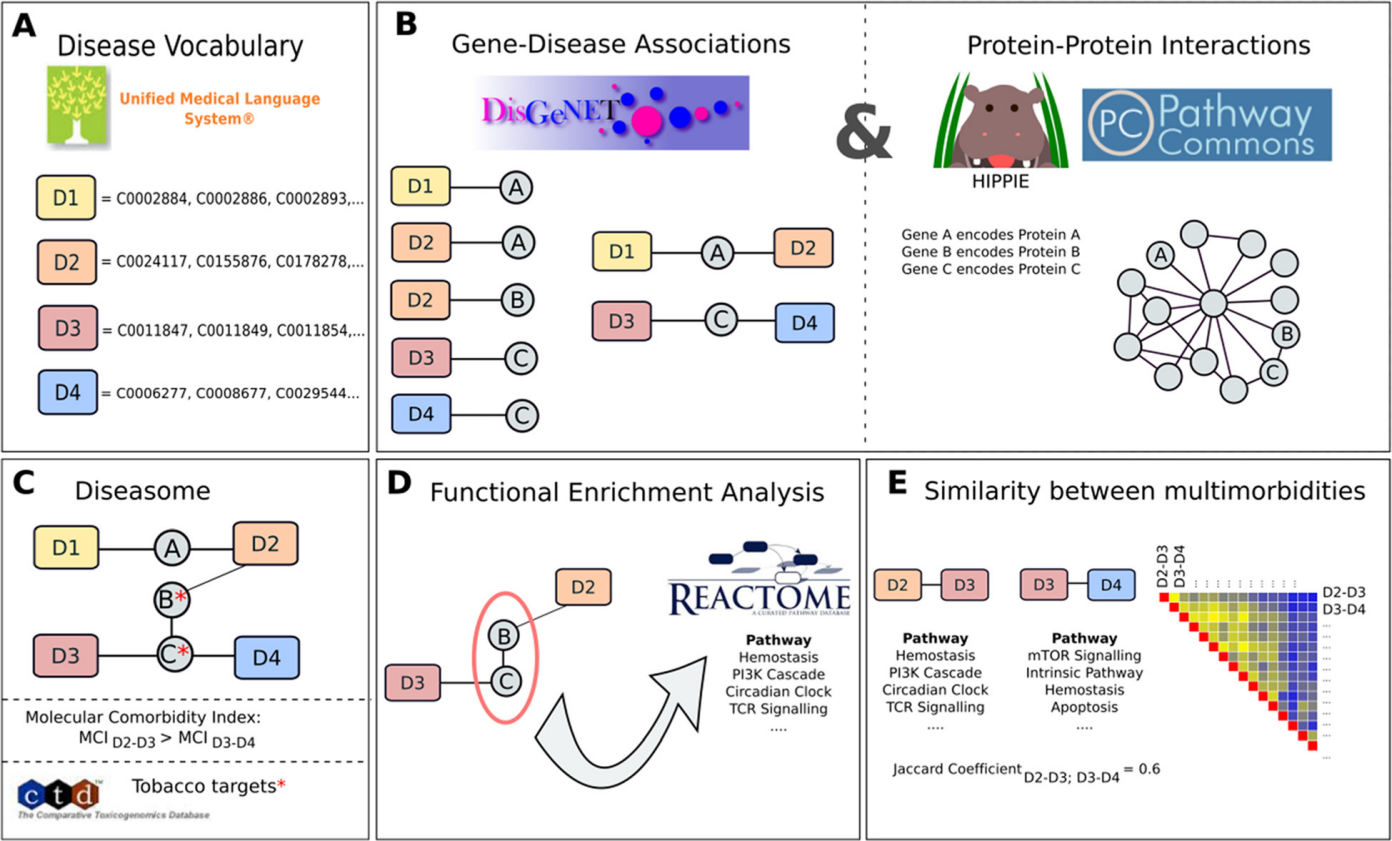
**Schematic representation of the methodology applied in this study.** *Panel* ***A***: Clinical experts selected prevalent multimorbidities in COPD, and manually curated lists of Concept Unique Identifiers (CUI) obtained from the Unified Medical Language System (UMLS) Metathesaurus to extract relevant synonyms for each disease. *Panel* ***B***: DisGeNet was used to obtain gene-disease associations and every protein encoded by the selected gene was mapped into a protein-protein network created from HIPPIE and Pathway Commons databases. *Panel* ***C***: Gene-disease associations and protein-protein interaction information were combined to build the diseasome. The Molecular Comorbidity Index estimates the strength of association between 2 diseases based on shared genes and proteins. The genes and proteins targeted by cigarette smoke components were obtained from CTD. *Panel* ***D***: A functional enrichment analysis was performed to identify the most likely pathways that could lead to the development of a specific multimorbidity and *Panel* ***E***: pathway similarity between multimorbidities was assessed calculating the Jaccard Coefficient.

Clinical experts selected 16 prevalent diseases frequently associated with COPD (Table 1) [12–14]. Their Concept Unique Identifiers (CUI) were obtained from the Unified Medical Language System (UMLS) Metathesaurus (Figure 1 panel A) [15]. After manual curation, the disease vocabulary used in the analysis included a mean of 59 CUIs (range 4–332) for each disease (and 14 CUIs for COPD, 33 for emphysema and 16 for chronic bronchitis (Additional file 1: Table S1)).

**Table 1:** **The diseases analyzed in this study and their corresponding number of UMLS Concept Unique Identifiers (CUIs) and associated genes in DisGeNET**

**Disease class**	**Disease**	**CUIs**	**Genes**
**COPD**			
Respiratory Tract Diseases	Chronic bronchitis	16	11
	COPD	14	181
	Emphysema	33	11
**COPD associated diseases**			
Nutritional and Metabolic Diseases	Diabetes	332	757
	Metabolic syndrome	4	47
	Obesity	47	313
Musculoskeletal Diseases	Muscle weakness	8	65
	Osteoporosis	37	128
Respiratory Tract Diseases	Pulmonary hypertension	22	57
	Lung cancer	81	1369
	Sleep apnea	19	27
Hemic and Lymphatic Diseases	Anemia	5	13
	Polycythemia	11	16
Cardiovascular Diseases	Atrial fibrillation	21	74
	Heart failure	73	410
	Ischemic heart disease	151	618
	Stroke	57	189
Pathological Conditions, Signs and Symptoms	Cachexia	8	19
Behavior and Behavior Mechanisms	Depression	105	160

Diseases are classified according the MeSH disease herarchy.

### Building the COPD diseasome

**Figure 2:**
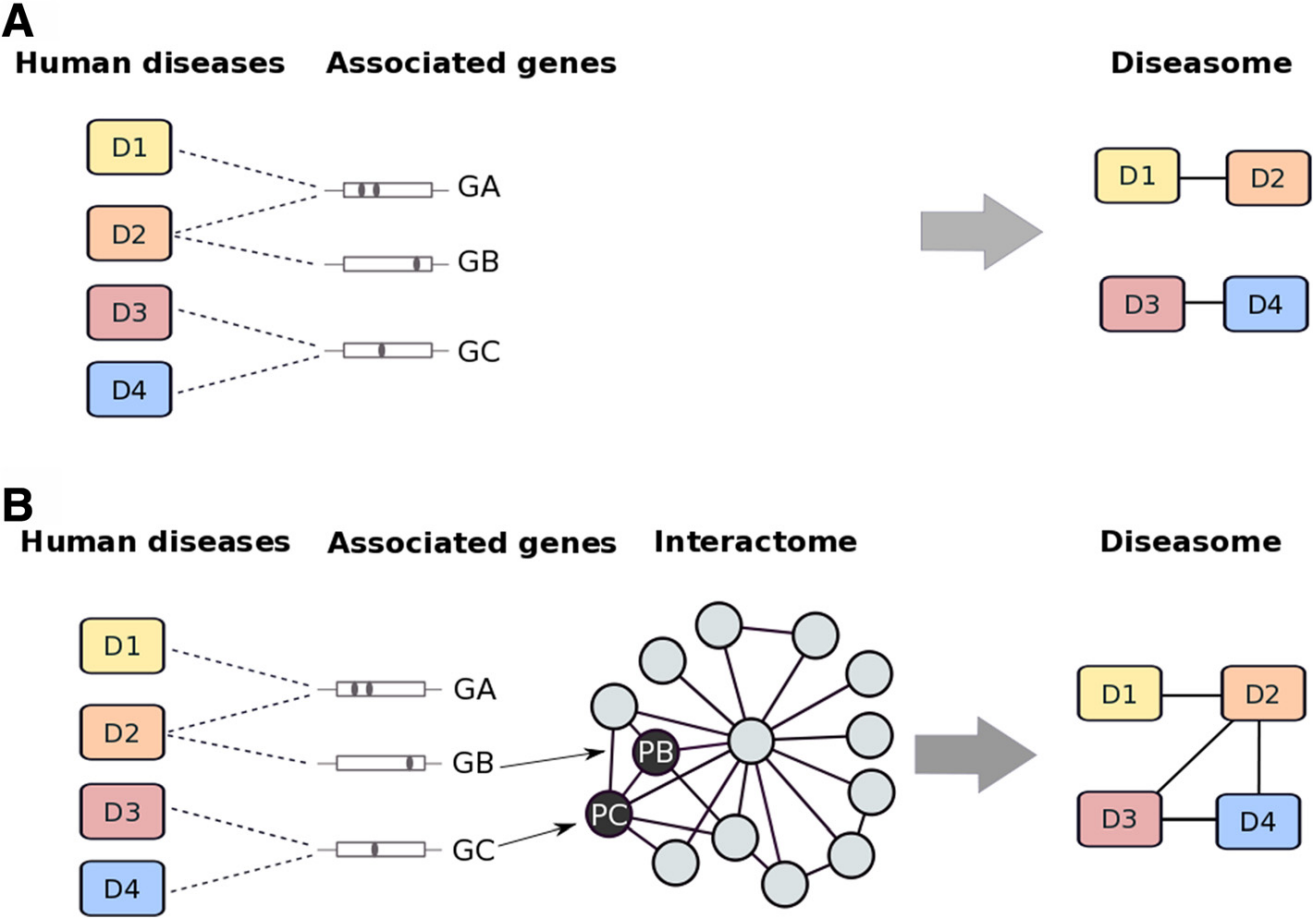
**Building the diseasome**. *Panel* ***A***: four different diseases (D1, D2, D3 and D4) can share relevant genes (GA, GB, GC) (left graph). Using this information, a diseasome can be built where D1 shares GA with D2, and D3 shares GC with D4 (right). *Panel* ***B***: The genetic information identified above can be complemented with the interactome to build the diseasome, where the protein encoded by GB (PB) interacts with that encoded by GC (PC) (left graph). For further explanations, see text.

The “diseasome” is a network representation of the associations between diseases, where two of them are linked if they share alterations in genes or proteins, or if the altered proteins are connected in the interactome (9) (Figure 2). DisGeNET (version 2.0), a database that integrates information on human diseases and their associated genes from various public databases and from the biomedical literature [16], was used to collect information on proteins altered in COPD and the multimorbidities studied here using our disease vocabulary (Figure 1 panel B). The human interactome was obtained by combining PPI information from the Pathway Commons database [17] (http://www.pathwaycommons.org/), gathering biological pathway information collected from public pathway databases and HIPPIE [18] (http://cbdm.mdc-berlin.de/tools/hippie/information.php), a human PPI database that integrates multiple experimental PPI datasets (Figure 1 panel B).

When building the diseasome based on proteins in common between two diseases, it could be argued that it is more likely to find shared proteins among diseases that have been characterized more extensively. In order to minimize this bias when estimating the strength of the association between two given diseases (dis1 and dis2) in the diseasome (Figure 1 panel C), we calculated the *Molecular Comorbidity Index* (MCI) as defined by:

$$ MC{I}_{dis1, dis2} = \frac{\left|\left( protein{s}_{dis1}{\displaystyle \cap }\  protein{s}_{dis2}\right)\ {\displaystyle \cup }\  protein{s}_{dis1\to dis2}{\displaystyle \cup }\  protein{s}_{dis2\to dis1}\right|}{\left| protein{s}_{dis1}{\displaystyle \cup }\  protein{s}_{dis2}\right|} $$

where *proteins*_*dis1*_ and *proteins*_*dis2*_ are the proteins associated with disease 1 and 2, respectively, *proteins*_*dis1→dis2*_ are those proteins associated with disease 1 that interact with those associated with disease 2 (and vice versa (*proteins*_*dis2→dis1*_)), ∩ is the intersection operator and ∪ is the union operator between two sets of elements (*proteins*_*dis1*_ and *proteins*_*dis2*_). The sets resulting in both numerator and denominator are written within vertical bars to indicate their cardinality (number of element).

### Functional analysis of multimorbidity proteins

To identify the most significant biological functions of the shared proteins between two diseases, a functional enrichment analysis with biological pathways from Reactome was performed [19]. The Reactome database contains information on genes, proteins and their participation in biological pathways. In our analysis, we used the R package ReactomePA, which uses the hypergeometric function to test the significance of annotations [20]. Significant annotations were those with *q-values ≤* 0.05 (the *q-value* corresponds to the false discovery rate (FDR), an adjusted equivalent to the standard statistical *p-value* incorporated in ReactomePA).

To evaluate the number of biological pathways shared by COPD multimorbidities, we used the *Jaccard coefficient*, which is defined as:

$$ Jaccard\kern0.5em  coefficien{t}_{multimorbidity1, multimorbidity2}\kern0.5em =\kern0.5em \frac{\left| pathway{s}_{multimorbidity1}\cap \right.\left. pathway{s}_{multimorbidity2}\right|}{\left| pathway{s}_{multimorbidity1}\cup \right.\left. pathway{s}_{multimorbidity2}\right|} $$

where *multimorbidity*_*1*_ and *multimorbidity*_*2*_ represent two pairs of COPD multimorbidities (for instance lung cancer-COPD and diabetes-COPD) whereas *pathways of multimorbidity*_*1*_ and *pathways of multimorbidity*_*2*_ represent the biological pathways in which the proteins associated with the pairs *multimorbidity*_*1*_ and *multimorbidity*_*2*_ participate respectively. The Jaccard Coefficient is a measure of the degree of similarity between two COPD multimorbidities (for instance lung cancer-COPD and diabetes-COPD) at the level of biological pathways. Results were visualized as heat-maps using Gitools [21].

### Exploration of the tobacco exposome

Using the Comparative Toxicogenomics Database (CTD), a database gathering information about gene/protein and chemical interactions, we investigated if the genes and proteins shared by COPD multimorbidities were potential biological targets for chemical compounds present in the tobacco smoke [22,23].

## Results

### COPD is linked through genes and proteins to its comorbid diseases

**Figure 3:**
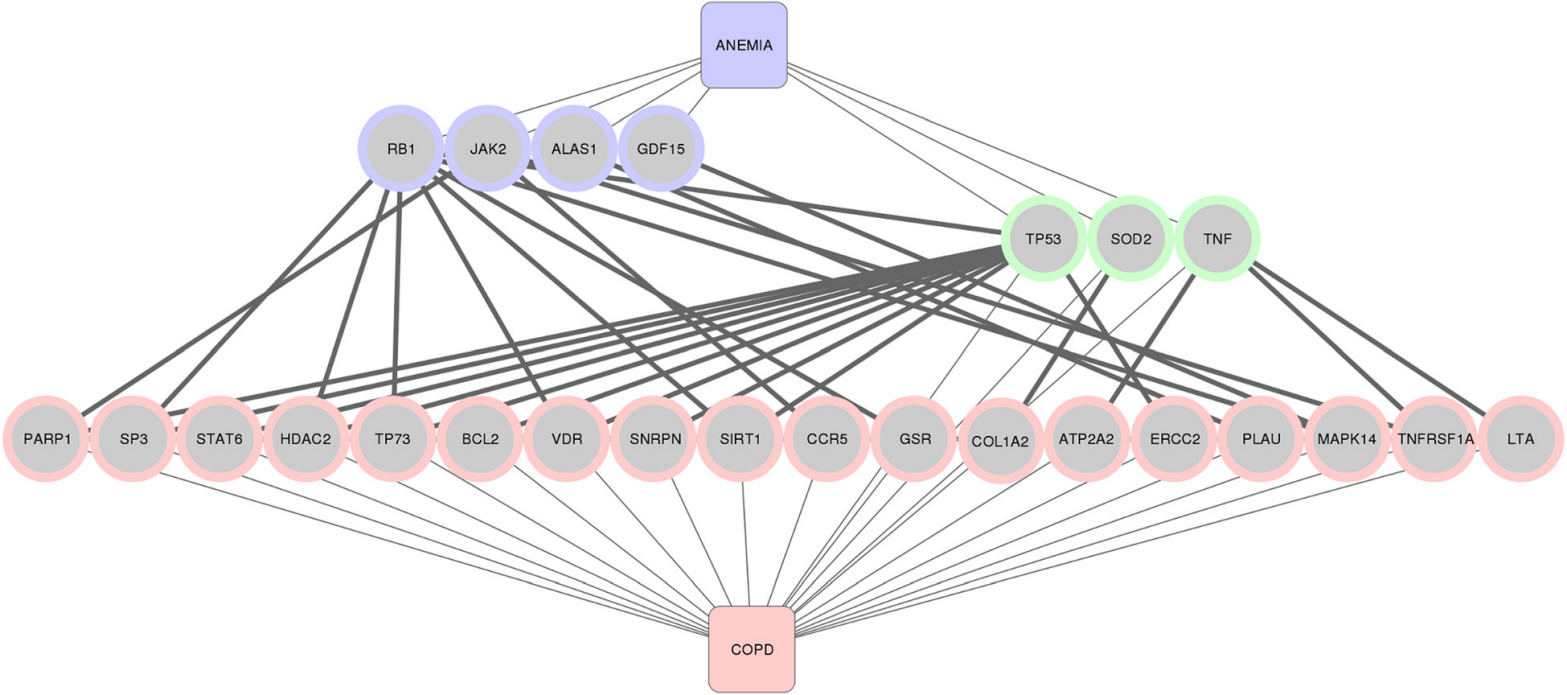
**COPD and anemia are connected at the molecular level.** The network represents the association between both diseases and proteins (thin edges) and the association between the proteins (thick edges). We show in green the proteins that are directly associated to both COPD and anemia (SOD2, TP53 and TNF). The pink and lila nodes are proteins associated to COPD or anemia, respectively, but also serve as bridges between both diseases because these proteins interact within the interactome.

COPD has been associated in the literature with alterations in 187 genes, whereas the number of genes associated with the 16 comorbidities analyzed here range from 19 (cachexia) to 1,369 (lung cancer) (Table 1 and Additional file 2: Figure S1 in the online data supplement). As explained in Figure 2, we used this knowledge on the genetic basis of these diseases in combination with data obtained from the interactome to build the COPD diseasome, and found that all 16 comorbidities studied here can be connected through shared genes and/or proteins that interact within cellular networks. Figure 3 illustrates how COPD and anemia are linked through this network of interactions. Both diseases are directly associated to 3 proteins in common, SOD2, TP53 and TNF (green nodes in Figure 3), and are also connected through the interactions between 22 proteins (pink and lila nodes in Figure 3). These bridging proteins constitute potential candidates to explain the association between COPD and anemia that should be further investigated.

**Figure 4:**
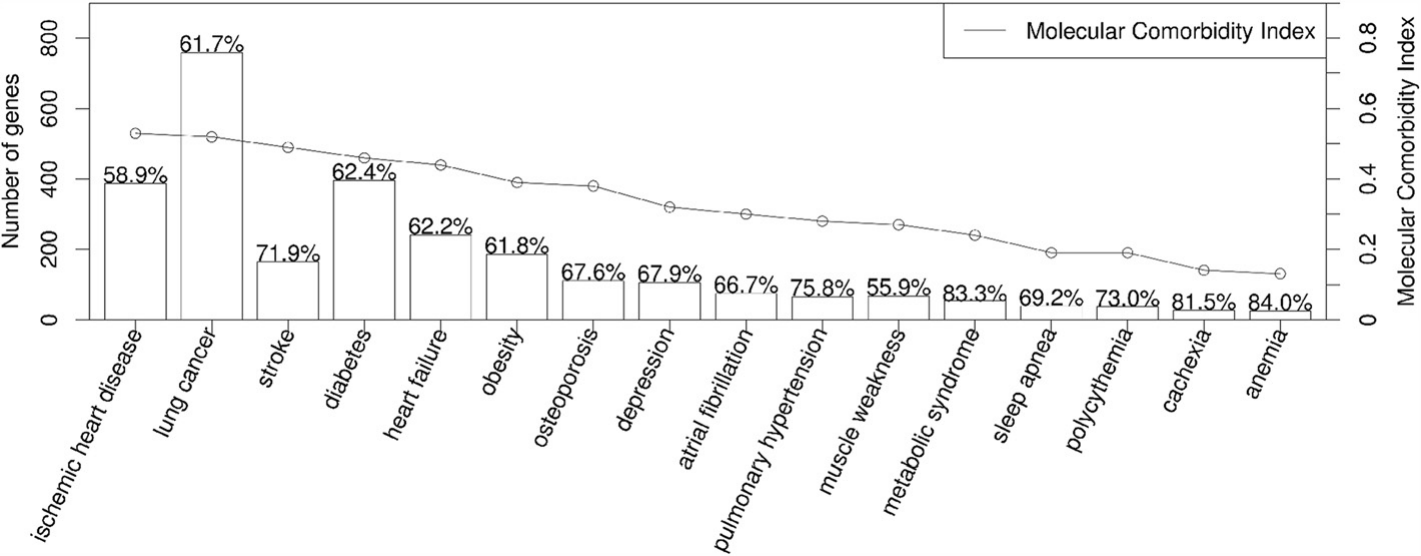
**Number of proteins shared between COPD and each of the multimorbidities studied here (bars) and their corresponding MCIs (line)**. The percentages shown on top of bars indicate the proportion of shared proteins targeted by, at least, one chemical compound present in tobacco smoke as per the CTD. For further explanations, see text.

In the COPD diseasome, the number of shared genes or proteins ranged from less than 100 (COPD and anemia) to more than 700 (COPD and lung cancer) (Figure 4). In terms of the Molecular Comorbidity Index (MCI), that is an indicator of the strength of the association between two diseases normalized for those better studied (for instance, lung cancer, see Methods), ischemic heart disease ranked first in the list of COPD multimorbidities (approximately 400 shared proteins with a MCI of 0.53), followed by lung cancer, stroke and diabetes. The frequency distribution of the MCI (Figure 4) paralleled that reported in the literature for the multimorbidities studied here [10,24,25].

The proposed network medicine workflow not only allows a global analysis of the genes and proteins supporting a particular multimorbidity (see next sections), but it also provides detailed information on specific genes and their association to the multimorbid diseases under study. For example, the gene ADRB2, is associated to several multimorbidities: COPD with cardiovascular diseases (atrial fibrillation, heart failure, ischemic heart disease and stroke), and with diabetes, lung cancer and obesity. Interestingly, the protein encoded by this gene has been shown to participate in smooth muscle relaxation in bronchi resulting in a facilitated respiration [26]; in blood vessels, it dilates coronary and skeletal muscle arteries [27] and it also contributes to insulin secretion from pancreas [28]. ADRB2 has been the focus of pharmacogenomics studies in COPD: several polymorphisms of this gene have been shown to influence patient response to bronchodilators treatment [29]. A recent study showed a significant correlation between the R16G polymorphism with COPD severity in term of FEV1 (forced expiratory volume in 1 second) in a Greek population [30]. Interestingly, the R16G–Q27E haplotype has been shown to be associated with glucose tolerance and insulin sensitivity in obese postmenopausal women [27]. Thus, this polymorphism might influence both bronchodilator response and glucose tolerance in specific patient populations, constituting a potential link to explain the association between COPD and diabetes.

Another study in a Danish population showed that the ADRB2 T164I polymorphism is associated with a reduced lung function and an increased risk of COPD in the general population [31]. This polymorphism is also associated with increased blood pressure, higher frequency of hypertension and increased risk of ischemic heart disease amongst women in the general population [26]. Thus, the ADRB2 T164I polymorphism could explain the association between COPD and cardiovascular diseases.

Further analysis using haplotype frequencies indicated that haplotypes G16-Q27-I164 and (non-G16-Q27)-T164, as compared to the reference haplotype G16-Q27-T164, were significantly associated with a decreased risk of myocardial infarction [32]. In summary, the ADRB2 gene represents an interesting candidate to explain the multimorbidity of COPD with diabetes, obesity and ischemic heart disease.

### Exploring the function of shared genes and proteins in the COPD diseasome

**Figure 5:**
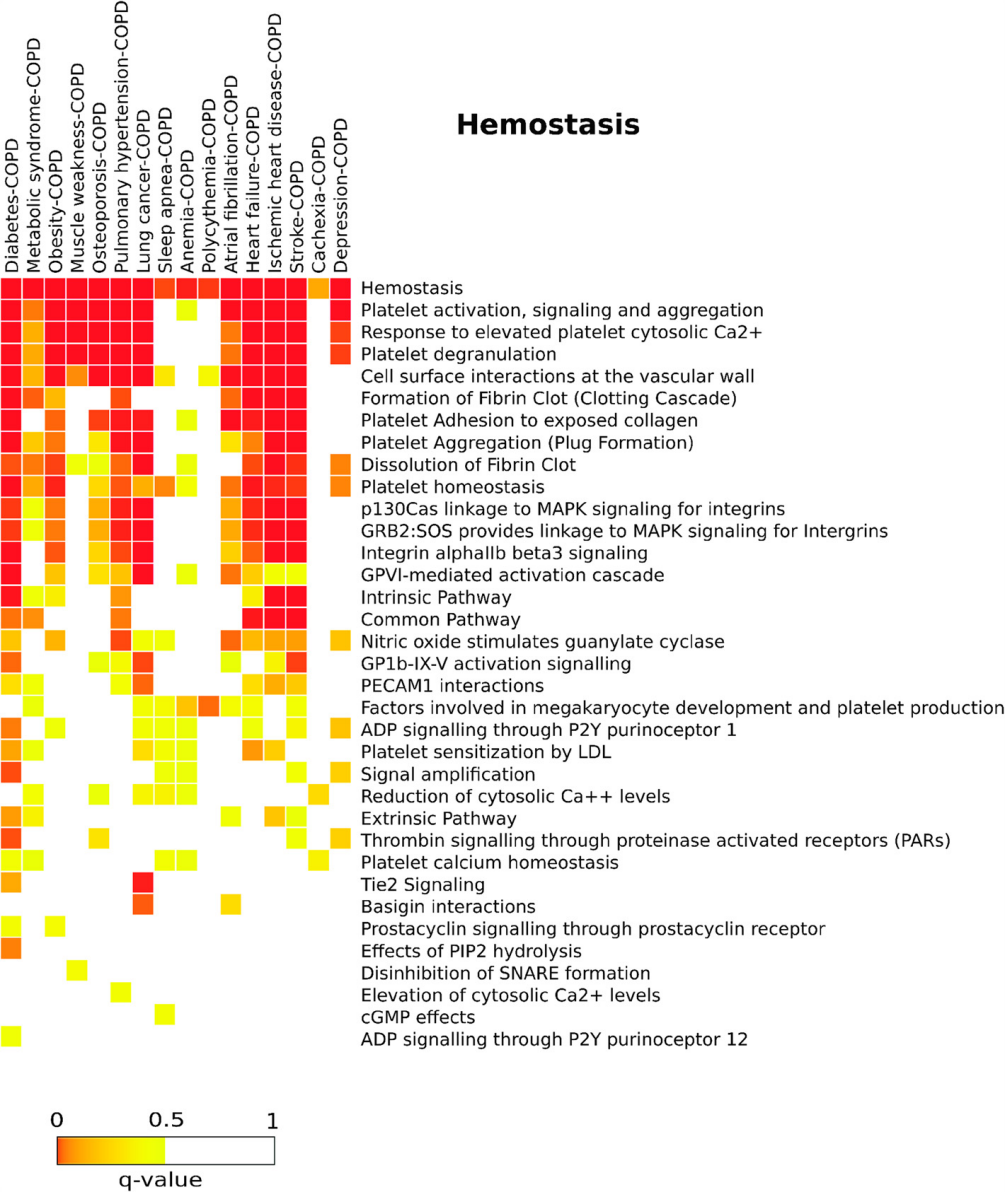
**Hemostasis Biological pathway for COPD multimorbidities**. The heat-map represents the enrichment of different pathways belonging to the Hemostasis category. Each cell of the heatmap is colored according to the q-value of the pathway enrichment for each COPD multimorbidity, where the most significant signals are from red (q-value ~ 0) to yellow (q-value < 5%). When a pathway is not statistically significant (q-value > 5%), the cell is left blank.

To identify the most relevant biological functions of the genes and proteins shared by the COPD diseasome, we performed a functional enrichment analysis with biological pathways from Reactome on the set of common proteins, as detailed in the Methods section. Significant annotations were those with *q-values ≤* 0.05, and results are visualized here as heat-maps. We observed that “Innate immune response” pathways were enriched in almost all of the multimorbidity pairs studied, particularly in those of COPD with diabetes, osteoporosis, lung cancer, heart failure or ischemic heart disease (Additional file 3: Figure S2 in the Supporting information). “Adaptive immune response” pathways showed a similar pattern: they were also enriched in all the pairs with the exception of those of COPD with metabolic syndrome, anemia, sleep apnea and cachexia (Additional file 4: Figure S3 in the online data supplement). Likewise, with the exception of the pair pulmonary hypertension-COPD, “Apoptosis” pathways and related signaling events, such as “TNF signaling” or “Death receptor signaling” were also involved in most multimorbidity pairs (Additional file 5: Figure S4 in the online data supplement). Interestingly, proteins involved in the “Nitric oxide metabolism” pathway were associated with COPD multimorbidities involving the cardiovascular system (atrial fibrillation, heart failure, ischemic heart disease and stroke), metabolic diseases (diabetes, metabolic syndrome), osteoporosis, pulmonary hypertension, lung cancer and depression (Additional file 6: Figure S5 in the online data supplement). “Hemostasis” pathways, including “Platelet activation, signaling and aggregation” were also enriched in all the pairs studied (Figure 5). Finally, “Cell cycle” pathways were enriched in lung cancer-COPD, diabetes-COPD and, surprisingly, in depression-COPD (Additional file 7: Figure S6 in the online data supplement).

### Overlap of identified biological pathways in the COPD diseasome

**Figure 6:**
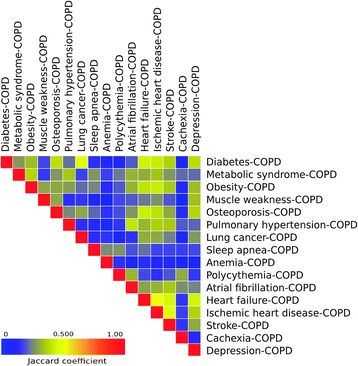
**Similarities between COPD multimorbidities.** Each cell is colored according to the value of the Jaccard coefficient that represents the degree of similarity between two COPD multimorbidities, considering biological pathways. For further explanations, see text.

To explore the degree of similarity between two COPD multimorbidities (for instance lung cancer-COPD and diabetes-COPD) at the level of biological pathways, we used the Jaccard coefficient (see Methods). We found the highest Jaccard coefficient values among pairs of COPD and several CVDs, including heart failure, ischemic heart disease and stroke, which share approximately 50% of the involved pathways (Figure 6). Likewise, the level of pathway coincidence was also considerable between pairs of CVD-COPD and pairs of COPD with nutritional and metabolic diseases. More surprisingly was the observation of a relatively high Jaccard coefficient between CVD-COPD pairs and osteoporosis-COPD, as well as that lung cancer-COPD and diabetes-COPD that showed a pathway similarity of 48% (lung cancer and diabetes without taking into account COPD showed only 35% of pathway similarity). Albeit to a lesser degree, nutritional and metabolic diseases-COPD and musculoskeletal diseases-COPD groups present pathway similarities as well. Other interesting associations that emerged from this analysis included the overlap between depression-COPD and diabetes-COPD, and between depression-COPD and heart failure-COPD. Finally, it was also of interest to note that the biological pathways involved in some comorbid disease-COPD pairs were almost unrelated to those of others (for instance, anemia-COPD, cachexia-COPD, sleep-apnea-COPD or polycythemia-COPD) (Figure 6). All in all, these observations highlight interesting associations between COPD multimorbidities at the biological pathway level.

### Exploration of the tobacco exposome

According to the CTD [33], the 106 tobacco smoke chemicals present in the tobacco smoke [22,23] target an average of 69% of the proteins shared by COPD and the multimorbidities shown in Figure 4 (see complete data set on Additional file 8: Table S2 in the online data supplement). For instance, 468 out of the 759 (61.7%) proteins shared by COPD and lung cancer are known targets of at least one compound present in tobacco smoke. This proportion was even higher (72%) in the case of stroke with COPD. This is not surprisingly since tobacco smoke is a main risk factor of COPD, CVD and lung cancer. Yet, even in multimorbidities that, at first glance, might seem unrelated to smoking, such as metabolic syndrome, diabetes or anemia, we also found a high percentage of shared proteins that are established targets of tobacco smoke compounds in the CTD, which provides potential candidates to investigate the role of tobacco smoke compounds in COPD multimorbidities.

## Discussion

The network medicine analysis of COPD multimorbidities presented here shows that 16 prevalent COPD multimorbidities often share genes, proteins and biological pathways with COPD, and that many of these proteins are targets for tobacco smoke chemicals. Overall, these observations support the *shared component hypothesis* of the COPD diseasome, and allow the identification of specific molecular links (genes, proteins, biological pathways) between COPD and its comorbid diseases.

The vast amount of information available today in different areas of science makes it necessary to properly mine and connect it in a network of information that allows the generation of new knowledge. This strategy has the potential to bring out new hypotheses that are not self-evident when using more traditional scientific approaches. In the present study, we performed an integrative mining approach developed to exploit information from different databases (HIPPIE, Pathway Commons, Reactome, CTD, DisGeNET) and experts in COPD multimorbidities (AF, RF, JR, BC, AA, JG) and bioinformatics (SG, JP, FS, LIF), who participated in the analysis phases. Future studies will have to explore experimentally the new hypotheses generated in this analysis.

COPD is a paradigmatic gene-environment disease since tobacco smoking, and other environmental factors, are well established risk factors, but not all exposed subjects develop the disease, suggesting a role for the genetic background of the exposed individual [34]. The fact that COPD clusters in families further supports this latter aspect [35]. Multimorbidities frequently occur in COPD patients and have a very significant impact in the natural history of the disease [34]. Yet, the precise pathogenic mechanism(s) underlying this COPD diseasome are unclear. To our knowledge, this is the first study to use a comprehensive and integrative bioinformatic approach to investigate the so-called *shared component hypothesis* [7–9] as a pathogenic mechanism of COPD multimorbidities.

Our findings suggest that the most prevalent COPD multimorbidities are related at the molecular level, and highlight some previously overlooked pathways that might contribute to explain their co-occurrence. In this context, that COPD shares certain molecular mechanisms with cardiovascular diseases or lung cancer is probably not surprising. By contrast, that it also shares these mechanisms with depression or diabetes is more intriguing. Likewise, it is of note that proteins involved in the “Nitric Oxide Metabolism” pathway were associated with multimorbidities involving the cardiovascular system, but also with metabolic diseases, as well as with osteoporosis, pulmonary hypertension, lung cancer and depression (Additional file 6: Figure S5 in the online data supplement).

Some of the shared biological pathways identified, including those related to the immune response, apoptosis, cytokine signaling or endothelial function, are well described in the field, albeit they serve as a proof of concept for the methodology employed. Yet, our analysis has also identified others pathways not previously related to COPD multimorbidities, such as hemostasis in multimorbidities other than cardiovascular disorders, or alterations in cell cycle in the COPD-depression association. Of note, it has been previously reported that increases in platelet activation and aggregation augment the risk of patients with depression to suffer CVD [35]. It is also worth noting that abnormalities in adult neurogenesis has been implicated in the etiology of depression [36,37] thus indicating that a disruption of cell cycle pathways in neural cells might underlie the observed association of COPD with depression. These new and unexpected findings require now to be tested experimentally but, all in all, our observations suggest that the multimorbidities in COPD should probably be understood as a network of clinical manifestations (i.e., diseasome) that expresses a complex interaction between a number of environmental, biological and genetic (and epigenetic) governing factors [2,3].

Tobacco smoke is the main risk factor of both COPD and some of its associated diseases [38]. According to CTD [33], chemicals contained in the tobacco smoke target an average of 69% of the proteins studied (Figure 3). This is not surprising for diseases like CVD or lung cancer, where tobacco smoking is a well-established risk factor, but it may open new avenues for research in other comorbid diseases.

### Strengths and limitations

The novelty, as well as the unbiased and integrative nature of our scientific approach, is a clear strength of our analysis. However, some limitations are worth noting. The most relevant one is that our analysis depends on the scientific information that is available and recorded in public databases, such as Medline, Reactome and other repositories of biological data. Further, due to limitations in database development and curation, many of such databases are likely to be incomplete [39]. Hence, the results of our analysis should be mostly regarded as hypothesis generators requiring experimental validation.

## Conclusions

Our network medicine analysis identified plausible molecular connections in the COPD diseasome (through genes, proteins and biological pathways), and showed that many of them are targets of the tobacco exposome, proposing new areas of research for understanding the molecular underpinning of COPD multimorbidities.

## Additional files

Additional file 1: Table S1. List of the UMLS CUIs for each disease represented in Table 1. Additional file 2: Figure S1. Venn diagram representing the genes associated to COPD, chronic bronchitis and emphysema, and the genes shared by these diseases. One hundred eighty seven genes are associated to the three diseases in the DisGeNET database [16]. Additional file 3: Figure S2. Innate Immune System Biological pathway for COPD multimorbidities. The heat-map represents the enrichment of different pathways belonging to the “Innate Immune System” category. Each cell of the heatmap is colored according to the q-value of the pathway enrichment for each COPD multimorbidity, where the most significant signals are from red (q-value ~ 0) to yellow (q-value < 5%). When a pathway is not statistically significant (q-value > 5%), the cell is left blank. Additional file 4: Figure S3. Adaptive Immune System Biological pathway for COPD multimorbidities. The heat-map represents the enrichment of different pathways belonging to the “Adaptive Immune System” category. Each cell of the heatmap is colored according to the q-value of the pathway enrichment for each COPD multimorbidity, where the most significant signals are from red (q-value ~ 0) to yellow (q-value < 5%). When a pathway is not statistically significant (q-value > 5%), the cell is left blank. Additional file 5: Figure S4. Apoptosis Biological pathway for COPD multimorbidities. The heat-map represents the enrichment of different pathways belonging to the “Apoptosis” category. Each cell of the heatmap is colored according to the q-value of the pathway enrichment for each COPD multimorbidity, where the most significant signals are from red (q-value ~ 0) to yellow (q-value < 5%). When a pathway is not statistically significant (q-value > 5%), the cell is left blank. Additional file 6: Figure S5. Nitric Oxide Metabolism Biological pathway for COPD multimorbidities. The heat-map represents the enrichment of different pathways belonging to the “Nitric Oxide Metabolism” category. Each cell of the heatmap is colored according to the q-value of the pathway enrichment for each COPD multimorbidity, where the most significant signals are from red (q-value ~ 0) to yellow (q-value < 5%). When a pathway is not statistically significant (q-value > 5%), the cell is left blank. Additional file 7: Figure S6. Cell Cycle Biological pathway for COPD multimorbidities. The heat-map represents the enrichment of different pathways belonging to the “Cell Cycle” category. Each cell of the heatmap is colored according to the q-value of the pathway enrichment for each COPD multimorbidity, where the most significant signals are from red (q-value ~ 0) to yellow (q-value < 5%). When a pathway is not statistically significant (q-value > 5%), the cell is left blank. Additional file 8: Table S2. List of tobacco smoke compounds used in this study and the genes that they target.

## Abbreviations

COPD: Chronic obstructive pulmonary disease
CTD: Comparative toxicogenomics database
CVD: Cardiovascular diseases
CUI: Concept unique identifier
MCI: Molecular comorbidity index
PPI: Protein-protein interactions
UMLS: Unified medical language system
