# Supplementary material for: Network medicine analysis of COPD multimorbidities
Source: Respir Res. 2014 Sep 24;15(1):111. doi: 10.1186/s12931-014-0111-4 (PMC4177421; doi:10.1186/s12931-014-0111-4)
Supplement: Additional file 1: Table S1. — List of the UMLS CUIs for each disease represented in Table 1. [file 12931_2014_111_MOESM1_ESM.pdf]

Category	UMLS CUI	UMLS name
Anemia	C0002884	Hypochromic anemia
Anemia	C0002886	Anemia, Macrocytic
Anemia	C0002893	Refractory anemias
Anemia	C0002896	Sideroblastic anemia
Anemia	C0541774	ANEMIA ACHRESTIC
Atrial fibrillation	C0004238	Atrial Fibrillation
Atrial fibrillation	C0004239	Atrial Flutter
Atrial fibrillation	C0155709	Atrial fibrillation and flutter
Atrial fibrillation	C0235480	Paroxysmal atrial fibrillation
Atrial fibrillation	C0340489	Lone atrial fibrillation
Atrial fibrillation	C0340490	Non-rheumatic atrial fibrillation
Atrial fibrillation	C0577699	Controlled atrial fibrillation
Atrial fibrillation	C0694539	Chronic atrial fibrillation
Atrial fibrillation	C0741281	atrial fibrillation new onset
Atrial fibrillation	C0741282	atrial fibrillation recurrent
Atrial fibrillation	C1142306	Atrial fibrillation with rapid ventricular response
Atrial fibrillation	C1281999	Rapid atrial fibrillation
Atrial fibrillation	C1542750	(Atrial fibrillation) or (atrial flutter)
Atrial fibrillation	C1834144	SICK SINUS SYNDROME 2, AUTOSOMAL DOMINANT
Atrial fibrillation	C1837014	ATRIAL FIBRILLATION, FAMILIAL, 3
Atrial fibrillation	C1837812	ATRIAL FIBRILLATION, FAMILIAL, 2
Atrial fibrillation	C1843687	ATRIAL FIBRILLATION, FAMILIAL, 1 (disorder)
Atrial fibrillation	C1862394	ATRIAL FIBRILLATION, FAMILIAL, 4
Atrial fibrillation	C1969099	ATRIAL FIBRILLATION, FAMILIAL, 5
Atrial fibrillation	C2585653	Persistent atrial fibrillation
Atrial fibrillation	C2586056	Permanent atrial fibrillation
Cachexia	C0006625	Cachexia
Cachexia	C0013911	Emaciation
Cachexia	C0043046	Wasting Syndrome
Cachexia	C0221405	Pituitary cachexia
Cachexia	C0235394	Wasting
Cachexia	C0242343	Panhypopituitarism
Cachexia	C0392513	Inanition
Cachexia	C0410282	Muscle cachexia
Chronic bronchitis	C0006277	Bronchitis
Chronic bronchitis	C0008677	Bronchitis, Chronic
Chronic bronchitis	C0029544	Other chronic bronchitis
Chronic bronchitis	C0155873	Mucopurulent chronic bronchitis
Chronic bronchitis	C0264322	Chronic tracheitis
Chronic bronchitis	C0264323	Chronic tracheobronchitis
Chronic bronchitis	C0264345	Smokers' cough
Chronic bronchitis	C0264346	Purulent chronic bronchitis
Chronic bronchitis	C0264348	Chronic asthmatic bronchitis
Chronic bronchitis	C0264404	Chronic allergic bronchitis
Chronic bronchitis	C0264422	Occupational bronchitis
Chronic bronchitis	C0348807	Mixed simple and mucopurulent chronic bronchitis
Chronic bronchitis	C0497359	Chronic bronchitis/bronchiectasis
Chronic bronchitis	C0856695	Acute exacerbation of chronic bronchitis
Chronic bronchitis	C1456131	Obstructive chronic bronchitis with acute bronchitis
Chronic bronchitis	C1535054	(Simple chronic bronchitis) or (smoker's cough) or (senile tracheobronchitis)
COPD	C0024117	Chronic Obstructive Airway Disease
COPD	C0155876	Obstructive chronic bronchitis with (acute) exacerbation
COPD	C0178278	CHRONIC OBSTRUCTIVE PULMONARY DISEASE AND ALLIED CONDITIONS
COPD	C0302378	Chronic airway obstruction, NEC in ICD9CM_2012
COPD	C0348693	Other specified chronic obstructive airways disease
COPD	C0348817	Chronic obstructive pulmonary disease with acute exacerbation, unspecified
COPD	C0348818	Chronic obstructive pulmonary disease with acute lower respiratory infection
COPD	C0600260	Lung Diseases, Obstructive
COPD	C0730604	Mild chronic obstructive pulmonary disease
COPD	C0730605	Moderate chronic obstructive pulmonary disease
COPD	C0730607	Severe chronic obstructive pulmonary disease

COPD	C0810017	Chronic obstructive pulmonary disease and bronchiectasis
COPD	C1847014	PULMONARY DISEASE, CHRONIC OBSTRUCTIVE, SEVERE EARLY-ONSET
Depression	C0001546	Adjustment Disorders
Depression	C0001723	Affective Disorders, Psychotic
Depression	C0011570	Mental Depression
Depression	C0011574	Involutional Depression
Depression	C0011579	Reactive depression
Depression	C0011580	Depression, Reactive, Psychotic
Depression	C0011581	Depressive disorder
Depression	C0024517	Major Depressive Disorder, Single Episode, Unspecified
Depression	C0025193	Melancholia
Depression	C0033958	Psychosis, Brief Reactive
Depression	C0085159	Seasonal Affective Disorder
Depression	C0154318	Arteriosclerotic dementia with depression
Depression	C0154403	Major Depressive Disorder, Single Episode, Mild
Depression	C0154404	Major Depressive Disorder, Single Episode, Moderate
Depression	C0154405	Major Depressive Disorder, Single Episode, Severe Without Psychotic Features
Depression	C0154406	Major Depressive Disorder, Single Episode, Severe With Psychotic Features
Depression	C0154408	Single major depressive episode, in full remission
Depression	C0154409	Major Depressive Disorder, Recurrent, Unspecified
Depression	C0154410	Major Depressive Disorder, Recurrent, Mild
Depression	C0154411	Major Depressive Disorder, Recurrent, Moderate
Depression	C0154412	Major Depressive Disorder, Recurrent, Severe Without Psychotic Features
Depression	C0154413	Major Depressive Disorder, Recurrent, Severe With Psychotic Features
Depression	C0154415	Major Depressive Disorder, Recurrent, In Full Remission
Depression	C0154437	Atypical depressive disorder
Depression	C0221480	Recurrent depression
Depression	C0221745	Depression suicidal
Depression	C0236653	Multi-infarct dementia with depression
Depression	C0236763	Major depression single episode, in partial remission
Depression	C0236764	Major Depressive Disorder, Recurrent, In Partial Remission
Depression	C0270455	Mild major depression
Depression	C0270456	Moderate major depression
Depression	C0270457	Severe major depression without psychotic features
Depression	C0270458	Severe major depression with psychotic features
Depression	C0270459	Severe major depression with psychotic features, mood-congruent
Depression	C0270460	Severe major depression with psychotic features, mood-incongruent
Depression	C0270461	Major depression in remission
Depression	C0270462	Major depression in partial remission
Depression	C0270463	Major depression in complete remission
Depression	C0270467	Severe major depression, single episode, with psychotic features, mood-congruent
Depression	C0270468	Severe major depression, single episode, with psychotic features, mood-incongruent
Depression	C0270470	Major depression, single episode, in complete remission
Depression	C0270475	Severe recurrent major depression with psychotic features, mood-congruent
Depression	C0270476	Severe recurrent major depression with psychotic features, mood-incongruent
Depression	C0270477	Recurrent major depression in remission
Depression	C0270488	Stuporous depression
Depression	C0282126	Depression, Neurotic
Depression	C0338629	Presenile dementia with depression
Depression	C0338631	Senile dementia with depression
Depression	C0338715	Drug-induced depressive state
Depression	C0338886	Major depressive disorder, single episode, in partial or unspecified remission
Depression	C0338890	Recurrent major depressive episodes, moderate
Depression	C0338893	Major depressive disorder, recurrent episode, in partial or unspecified remission
Depression	C0338897	Masked depression
Depression	C0338908	Mixed anxiety and depressive disorder
Depression	C0349217	Depressive episode, unspecified
Depression	C0349218	Recurrent depressive disorder
Depression	C0520665	Menopausal depression
Depression	C0553816	Endogenous depression - recurrent
Depression	C0556016	[X] Single episode agitated depression without psychotic symptoms
Depression	C0556017	[X] Single episode major depression without psychotic symptoms

Depression	C0581391	Chronic depression
Depression	C0588006	Mild depression
Depression	C0588007	Moderate depression
Depression	C0588008	Severe depression
Depression	C0679108	emotional and psychiatric depression
Depression	C0743072	depressive psychosis
Depression	C0743073	depression anxiety disorder
Depression	C0743076	depression psychotic feature
Depression	C0865313	Endogenous depression, single episode or unspecified
Depression	C0865316	Monopolar depression, single episode or unspecified
Depression	C0865317	Psychotic depression, single episode or unspecified
Depression	C0865364	Hyposomnia, insomnia or sleeplessness associated with depression
Depression	C0865365	Hyposomnia, insomnia or sleeplessness associated with major depression
Depression	C0865366	Hyposomnia, insomnia or sleeplessness associated with minor depression
Depression	C0865369	Hypersomnia associated with depression
Depression	C0865370	Hypersomnia associated with major depression
Depression	C0865371	Hypersomnia associated with minor depression
Depression	C0865401	Adjustment reaction with anxiety and depression
Depression	C0878684	SHORT syndrome
Depression	C1269683	Major Depressive Disorder
Depression	C1282644	Major depression, melancholic type
Depression	C1395188	depression; endogenous, with psychotic symptoms
Depression	C1524032	Depression and Suicide
Depression	C1534456	[X]Mixed anxiety and depressive disorder (& mild anxiety depression)
Depression	C1535539	Depression: [reactive (neurotic)] or [postnatal]
Depression	C1535540	(Neurotic depression reactive type) or (postnatal depression)
Depression	C1540574	[X] Depression: [other episodes] or [atypical] or [single episode masked NOS]
Depression	C1540578	[X]Recurrent depressive disorder (& [episodes of depressive reaction] or [episodes of psychogenic depression] or [episodes of reactive depression] or [seasonal depressive disorder])
Depression	C1540579	[X] Depression recurrent: [unspecified] or [monopolar NOS]
Depression	C1541764	Recurrent depression: [major episode] or [endogenous]
Depression	C1542445	[X] Severe depressive episode without psychotic symptoms: (& [single episode agitated depression] or [single episode major depression] or [single episode vital depression])
Depression	C1578538	[X]Depression without psychotic symptoms: [recurrent: [major] or [manic-depressive psychosis, depressed type] or [vital] or [current severe episode]] or [endogenous]
Depression	C1578541	[X]Vital depression, recurrent without psychotic symptoms
Depression	C1578542	[X]Endogenous depression without psychotic symptoms
Depression	C1579088	[X]Mild anxiety depression
Depression	C1579864	[X]Single episode of masked depression NOS
Depression	C1579865	[X]Recurrent episodes of psychogenic depression
Depression	C1579866	[X]Monopolar depression NOS
Depression	C1837529	MAJOR DEPRESSIVE DISORDER 2
Depression	C1837929	MAJOR DEPRESSIVE DISORDER 1
Depression	C1998428	Drug-induced mood disorder
Depression	C2004268	Depression: [single major episode] or [agitated] or [endogenous (including first episode)]
Depression	C2063866	Depressive Disorder, Treatment-Resistant
Depression	C2362914	clinical depression
Depression	C2700639	[X] (Depression: [episode, unspecified] or [NOS (& reactive)] or [depressive disorder NOS]
Diabetes	C0011847	Diabetes
Diabetes	C0011849	Diabetes Mellitus
Diabetes	C0011854	Diabetes Mellitus, Insulin-Dependent
Diabetes	C0011859	Lipoatrophic Diabetes Mellitus
Diabetes	C0011860	Diabetes Mellitus, Non-Insulin-Dependent
Diabetes	C0011870	Diabetes with other coma
Diabetes	C0011871	Diabetic peripheral angiopathy
Diabetes	C0011875	Diabetic Angiopathies
Diabetes	C0011877	Diabetic coma with ketoacidosis
Diabetes	C0011880	Diabetic Ketoacidosis
Diabetes	C0011881	Diabetic Nephropathy
Diabetes	C0011882	Diabetic Neuropathies
Diabetes	C0011884	Diabetic Retinopathy
Diabetes	C0017980	Glycosuria, Renal
Diabetes	C0020456	Hyperglycemia
Diabetes	C0020457	Hyperglycemic Hyperosmolar Nonketotic Coma
Diabetes	C0021655	Insulin Resistance

Diabetes	C0032969	Pregnancy in Diabetics
Diabetes	C0152025	Polyneuropathy
Diabetes	C0154181	NIDDM with peripheral circulatory disorder
Diabetes	C0154182	IDDM with peripheral circulatory disorder
Diabetes	C0154183	Diabetes with other specified manifestations
Diabetes	C0154830	Proliferative diabetic retinopathy
Diabetes	C0205734	Diabetes, Autoimmune
Diabetes	C0235398	DIABETES MELLITUS AGGRAVATED
Diabetes	C0235399	Diabetes mellitus reactivated
Diabetes	C0238086	DIABETES MELLITUS, TRANSIENT, OF NEWBORN
Diabetes	C0241861	DIABETES, STABLE
Diabetes	C0241862	DIABETES, TRUE RENAL
Diabetes	C0259741	Diabetes mellitus, latent
Diabetes	C0262448	Diabetes mellitus, chemical
Diabetes	C0271635	Diabetes mellitus without complication
Diabetes	C0271636	Insulin dependent diabetes mellitus type IA
Diabetes	C0271637	Insulin dependent diabetes mellitus type IB
Diabetes	C0271638	Diabetes mellitus type 2 in obese
Diabetes	C0271640	Secondary diabetes mellitus
Diabetes	C0271641	Malnutrition related diabetes mellitus
Diabetes	C0271642	Fibrocalculous pancreatic diabetes
Diabetes	C0271645	Diabetes mellitus associated with hormonal etiology
Diabetes	C0271646	Drug-induced diabetes mellitus
Diabetes	C0271648	Diabetes mellitus associated with receptor abnormality
Diabetes	C0271649	Diabetes mellitus associated with unlisted condition
Diabetes	C0271650	Impaired glucose tolerance
Diabetes	C0271680	Diabetic Polyneuropathies
Diabetes	C0271686	Diabetic Autonomic Neuropathy
Diabetes	C0271690	Insulin-resistant diabetes mellitus AND acanthosis nigricans
Diabetes	C0271693	Acquired generalized lipodystrophy
Diabetes	C0271701	Diabetes mellitus due to insulin receptor antibodies
Diabetes	C0339470	Visually threatening diabetic retinopathy
Diabetes	C0339473	Preproliferative diabetic retinopathy
Diabetes	C0339475	Proliferative diabetic retinopathy with new vessels elsewhere than on disc
Diabetes	C0341893	Diabetes mellitus in mother complicating pregnancy, childbirth AND/OR puerperium
Diabetes	C0341894	Diabetes mellitus in the pueperium - baby delivered during previous episode of care
Diabetes	C0341896	Diabetes mellitus in the puerperium - baby delivered during current episode of care
Diabetes	C0341897	Diabetes mellitus during pregnancy - baby delivered
Diabetes	C0341898	Diabetes mellitus - unspecified whether during pregnancy or the puerperium
Diabetes	C0342245	Diabetic oculopathy
Diabetes	C0342257	Complications of Diabetes Mellitus
Diabetes	C0342260	Diabetes mellitus with other specified manifestation
Diabetes	C0342262	Diabetes mellitus, adult onset, with other specified manifestation
Diabetes	C0342265	Diabetes mellitus, adult onset, with no mention of complication
Diabetes	C0342266	Insulin-treated non-insulin-dependent diabetes mellitus
Diabetes	C0342267	Malnutrition-related diabetes mellitus - fibrocalculous
Diabetes	C0342268	Protein-deficient diabetes mellitus
Diabetes	C0342269	Steroid-induced diabetes
Diabetes	C0342270	Diabetes mellitus associated with pancreatic disease
Diabetes	C0342271	Secondary endocrine diabetes mellitus
Diabetes	C0342274	Diabetes mellitus associated with genetic syndrome
Diabetes	C0342277	Diabetes mellitus autosomal dominant type II (disorder)
Diabetes	C0342280	Acrorenal field defect, ectodermal dysplasia, and lipoatrophic diabetes
Diabetes	C0342288	Insulin-dependent diabetes mellitus secretory diarrhea syndrome
Diabetes	C0342290	Abnormal metabolic state in diabetes mellitus
Diabetes	C0342294	Ketoacidosis in type I diabetes mellitus
Diabetes	C0342295	Ketoacidosis in type II diabetes mellitus
Diabetes	C0342302	Brittle diabetes
Diabetes	C0342312	Hypoglycemic event in diabetes
Diabetes	C0342313	Hypoglycemic state in diabetes
Diabetes	C0342335	Insulin resistance in diabetes
Diabetes	C0348447	Other specified diabetes mellitus

Diabetes	C0348448	Malnutrition-related diabetes mellitus with other specified complications
Diabetes	C0348449	Malnutrition-related diabetes mellitus with unspecified complications
Diabetes	C0348450	Unspecified diabetes mellitus with renal complications
Diabetes	C0348913	Insulin-dependent diabetes mellitus with renal complications
Diabetes	C0348914	Insulin-dependent diabetes mellitus with ophthalmic complications
Diabetes	C0348915	Insulin-dependent diabetes mellitus with neurological complications
Diabetes	C0348916	Multiple complications of type I diabetes mellitus
Diabetes	C0348917	Pre-existing diabetes mellitus, insulin-dependent
Diabetes	C0348918	Non-insulin-dependent diabetes mellitus with renal complications
Diabetes	C0348919	Diabetic oculopathy associated with type II diabetes mellitus
Diabetes	C0348920	Non-insulin-dependent diabetes mellitus with neurological complications
Diabetes	C0348921	Pre-existing diabetes mellitus, non-insulin-dependent
Diabetes	C0348922	Malnutrition-related diabetes mellitus with coma
Diabetes	C0348924	Malnutrition-related diabetes mellitus with renal complications
Diabetes	C0348925	Ophthalmic complication of malnutrition-related diabetes mellitus
Diabetes	C0348926	Malnutrition-related diabetes mellitus with neurological complications
Diabetes	C0348927	Malnutrition-related diabetes mellitus with peripheral circulatory complications
Diabetes	C0348928	Malnutrition-related diabetes mellitus with multiple complications
Diabetes	C0348929	Malnutrition-related diabetes mellitus without complications
Diabetes	C0348930	Pre-existing malnutrition-related diabetes mellitus
Diabetes	C0348931	Other specified diabetes mellitus with other specified complications
Diabetes	C0348932	Other specified diabetes mellitus with unspecified complications
Diabetes	C0348933	Other specified diabetes mellitus with coma
Diabetes	C0348934	Other specified diabetes mellitus with renal complications
Diabetes	C0348935	Other specified diabetes mellitus with ophthalmic complications
Diabetes	C0348936	Other specified diabetes mellitus with neurological complications
Diabetes	C0348937	Other specified diabetes mellitus with peripheral circulatory complications
Diabetes	C0348938	Other specified diabetes mellitus with multiple complications
Diabetes	C0348939	Unspecified diabetes mellitus with multiple complications
Diabetes	C0348941	Other specified diabetes mellitus with ketoacidosis
Diabetes	C0349363	Type II diabetes mellitus with multiple complications
Diabetes	C0362046	Prediabetes syndrome
Diabetes	C0375113	type II diabetes mellitus [non-insulin dependent type] [NIDDM type] [adult-onset type] or unspecified type, not stated as uncontrolled, without mention of complication
Diabetes	C0375114	type I diabetes mellitus [insulin dependent type] [IDDM] [juvenile type], not stated as uncontrolled, without mention of complication
Diabetes	C0375115	type II diabetes mellitus [non-insulin dependent type] [NIDDM type] [adult-onset type] or unspecified type, uncontrolled, without mention of complication
Diabetes	C0375116	type I diabetes mellitus [juvenile type], uncontrolled, without mention of complication
Diabetes	C0375117	type II diabetes mellitus [non-insulin dependent type] [NIDDM type] [adult-onset type] or unspecified type, not stated as uncontrolled, with ketoacidosis
Diabetes	C0375118	type I diabetes mellitus [insulin dependent type] [IDDM] [juvenile type], not stated as uncontrolled, with ketoacidosis
Diabetes	C0375119	type II diabetes mellitus [non-insulin dependent type] [NIDDM type] [adult-onset type] or unspecified type, uncontrolled, with ketoacidosis
Diabetes	C0375120	type I diabetes mellitus [juvenile type], uncontrolled, with ketoacidosis
Diabetes	C0375121	Diabetes mellitus with hyperosmolarity
Diabetes	C0375122	Diabetes mellitus, type II [non-insulin dependent type] [NIDDM type] [adult-onset type] or unspecified type with hyperosmolarity, not stated as uncontrolled
Diabetes	C0375123	Diabetes mellitus, type I [insulin dependent type] [IDDM] [juvenile type] with hyperosmolarity, not stated as uncontrolled
Diabetes	C0375124	Diabetes mellitustype II [non-insulin dependent type] [NIDDM type] [adult-onset type] or unspecified type with hyperosmolarity, uncontrolled
Diabetes	C0375125	Diabetes mellitus, type I [juvenile type] with hyperosmolarity, uncontrolled
Diabetes	C0375126	Diabetes mellitus, type II [non-insulin dependent type] [NIDDM type] [adult-onset type] or unspecified type, not stated as uncontrolled
Diabetes	C0375127	Diabetes mellitus, type I [insulin dependent type] [IDDM] [juvenile type], not stated as uncontrolled
Diabetes	C0375128	Diabetes mellitus with other coma, type II [non-insulin dependent type] [NIDDM type] [adult-onset type] or unspecified type, uncontrolled
Diabetes	C0375129	Diabetes mellitus with other coma, type I [juvenile type], uncontrolled
Diabetes	C0375130	Diabetes mellitus type II [non-insulin dependent type] [NIDDM type] [adult-onset type] or unspecified type, not stated as uncontrolled, with renal manifestations
Diabetes	C0375131	Diabetes mellitus type I [insulin dependent type] [IDDM] [juvenile type], not stated as uncontrolled, with renal manifestations
Diabetes	C0375132	Diabetes mellitus type II [non-insulin dependent type] [NIDDM type] [adult-onset type] or unspecified type, uncontrolled, with renal manifestations
Diabetes	C0375133	Diabetes mellitus type I [juvenile type], uncontrolled, with renal manifestations
Diabetes	C0375134	Diabetes mellitus type II [non-insulin dependent type] [NIDDM type] [adult-onset type] or unspecified type, not stated as uncontrolled, with ophthalmic manifestations
Diabetes	C0375135	Diabetes mellitus type I [insulin dependent type] [IDDM] [juvenile type], not stated as uncontrolled, with ophthalmic manifestations
Diabetes	C0375136	Diabetes mellitus type I [juvenile type], uncontrolled, with ophthalmic manifestations
Diabetes	C0375137	Diabetes mellitus type II [non-insulin dependent type] [NIDDM type] [adult-onset type] or unspecified type, not stated as uncontrolled, with neurological manifestations
Diabetes	C0375138	Diabetes mellitus type I [insulin dependent type] [IDDM] [juvenile type], not stated as uncontrolled, with neurological manifestations
Diabetes	C0375139	Diabetes mellitus type II [non-insulin dependent type] [NIDDM type] [adult-onset type] or unspecified type, uncontrolled, with neurological manifestations
Diabetes	C0375140	Diabetes mellitus type I [juvenile type], uncontrolled, with neurological manifestations
Diabetes	C0375141	Diabetes mellitus type II [non-insulin dependent type] [NIDDM type] [adult-onset type] or unspecified type, not stated as uncontrolled, with peripheral circulatory disorders
Diabetes	C0375142	Diabetes mellitus type I [insulin dependent type] [IDDM] [juvenile type], not stated as uncontrolled, with peripheral circulatory disorders

Diabetes	C0375143	Diabetes mellitus type II [non-insulin dependent type] [NIDDM type] [adult-onset type] or unspecified type, uncontrolled, with peripheral circulatory disorders
Diabetes	C0375144	Diabetes mellitus type I [juvenile type], uncontrolled, with peripheral circulatory disorders
Diabetes	C0375145	Diabetes mellitus type II [non-insulin dependent type] [NIDDM type] [adult-onset type] or unspecified type, not stated as uncontrolled, with other specified manifestations
Diabetes	C0375146	Diabetes mellitus type I [insulin dependent type] [IDDM] [juvenile type], not stated as uncontrolled, with other specified manifestations
Diabetes	C0375147	Diabetes mellitus type II [non-insulin dependent type] [NIDDM type] [adult-onset type] or unspecified type, uncontrolled, with other specified manifestations
Diabetes	C0375148	Diabetes mellitus type I [juvenile type], uncontrolled, with other specified manifestations
Diabetes	C0375149	Diabetes mellitus type II [non-insulin dependent type] [NIDDM type] [adult-onset type] or unspecified type, not stated as uncontrolled, with unspecified complication
Diabetes	C0375150	Diabetes mellitus type I [insulin dependent type] [IDDM] [juvenile type], not stated as uncontrolled, with unspecified complication
Diabetes	C0375151	Diabetes mellitus type II [non-insulin dependent type] [NIDDM type] [adult-onset type] or unspecified type, uncontrolled, with unspecified complication
Diabetes	C0375152	Diabetes mellitus type I [insulin dependent type] [IDDM] [juvenile type], uncontrolled, with unspecified complication
Diabetes	C0376128	Diabetes mellitus type II [non-insulin dependent type] [NIDDM type] [adult-onset type] or unspecified type, uncontrolled, with ophthalmic manifestations
Diabetes	C0403518	Microalbuminuric diabetic nephropathy
Diabetes	C0403519	Proteinuric diabetic nephropathy
Diabetes	C0406681	Soft tissue complication of diabetes mellitus
Diabetes	C0442862	Kimmelstiel Wilson kidney
Diabetes	C0477716	Glomerular disorders in diabetes mellitus
Diabetes	C0477821	Pre-existing diabetes mellitus, unspecified
Diabetes	C0494284	Type I diabetes mellitus without complication
Diabetes	C0494290	Type II diabetes mellitus without complication
Diabetes	C0494291	Malnutrition-related diabetes mellitus with ketoacidosis
Diabetes	C0554436	Diabetic gangrene
Diabetes	C0554876	poorly controlled diabetes mellitus
Diabetes	C0559093	Diabetes with other complications
Diabetes	C0564720	Diabetes with ketoacidosis - no coma
Diabetes	C0567407	Brittle type I diabetes mellitus
Diabetes	C0579065	Type I diabetes mellitus with ulcer
Diabetes	C0579066	Type II diabetes mellitus with ulcer
Diabetes	C0579119	Type I diabetes mellitus with gangrene
Diabetes	C0579120	Type I diabetes mellitus with retinopathy
Diabetes	C0579121	Type I diabetes mellitus - poor control
Diabetes	C0579122	Type II diabetes mellitus with gangrene
Diabetes	C0579123	Type II diabetes mellitus with retinopathy
Diabetes	C0579124	Type II diabetes mellitus - poor control
Diabetes	C0581383	Type I diabetes mellitus maturity onset
Diabetes	C0589117	Steroid-induced diabetes mellitus without complication
Diabetes	C0743125	Insulin resistant diabetes mellitus
Diabetes	C0810250	Diabetes with ketoacidosis or uncontrolled diabetes
Diabetes	C0810251	Diabetes with circulatory manifestations
Diabetes	C0810252	Diabetes with other manifestations
Diabetes	C0837004	Type 1 diabetes mellitus with diabetic cataract
Diabetes	C0837040	Type 2 diabetes mellitus with diabetic cataract
Diabetes	C0851207	Pre-existing diabetes mellitus, Type 1, in pregnancy
Diabetes	C0851208	Pre-existing diabetes mellitus, Type 2, in pregnancy
Diabetes	C0851209	Pre-existing malnutrition-related diabetes mellitus, predominantly related to pregnancy
Diabetes	C0851217	Pre-existing diabetes mellitus, unspecified, in pregnancy
Diabetes	C0853897	Diabetic Cardiomyopathies
Diabetes	C0854110	Insulin resistant diabetes
Diabetes	C0856873	borderline diabetes
Diabetes	C0865162	Diabetes mellitus without mention of complication or manifestation
Diabetes	C1263959	Diabetic ketoacidosis without coma
Diabetes	C1273344	Hyperosmolar non-ketotic state in type 2 diabetes mellitus
Diabetes	C1279301	Type I diabetes mellitus with mononeuropathy
Diabetes	C1279302	Type I diabetes mellitus with polyneuropathy
Diabetes	C1279303	Type I diabetes mellitus with nephropathy
Diabetes	C1279304	Type I diabetes mellitus with hypoglycemic coma
Diabetes	C1279305	Type I diabetes mellitus with peripheral angiopathy
Diabetes	C1279306	Type I diabetes mellitus with arthropathy
Diabetes	C1279307	Type I diabetes mellitus with neuropathic arthropathy
Diabetes	C1279309	Type II diabetes mellitus with mononeuropathy
Diabetes	C1279310	Type II diabetes mellitus with polyneuropathy
Diabetes	C1279311	Diabetes mellitus, adult onset, with renal manifestation
Diabetes	C1279312	Type II diabetes mellitus with hypoglycemic coma

Diabetes	C1279313	Type II diabetes mellitus with peripheral angiopathy
Diabetes	C1279314	Type II diabetes mellitus with arthropathy
Diabetes	C1279315	Type II diabetes mellitus with neuropathic arthropathy
Diabetes	C1282941	Diabetic neuropathy with neurologic complication
Diabetes	C1282951	Diabetes mellitus type 2 in nonobese
Diabetes	C1299614	Diabetes mellitus, adult onset, with unspecified complication
Diabetes	C1299632	Diabetic skin ulcer
Diabetes	C1303097	Diabetes mellitus with persistent microalbuminuria
Diabetes	C1303098	Diabetes mellitus with persistent proteinuria
Diabetes	C1303111	Type 1 diabetes mellitus with persistent proteinuria
Diabetes	C1303112	Type 1 diabetes mellitus with persistent microalbuminuria
Diabetes	C1303113	Type 2 diabetes mellitus with persistent proteinuria
Diabetes	C1303114	Type 2 diabetes mellitus with persistent microalbuminuria
Diabetes	C1318605	Diabetes mellitus induced by non-steroid drugs
Diabetes	C1318685	Type 1 diabetes mellitus with exudative maculopathy
Diabetes	C1319383	Type 2 diabetes mellitus with exudative maculopathy
Diabetes	C1446291	Diabetes mellitus induced by non-steroid drugs without complication
Diabetes	C1456657	Diabetic Nerve Problems
Diabetes	C1534918	Diabetes + neuropathy (& [amyotrophy])
Diabetes	C1535389	Diabetes mellitus: [adult onset, with no mention of complication] or [maturity onset] or [non-insulin dependent]
Diabetes	C1535390	Diabetes + nephropathy (& [Kimmelstiel-Wilson syndrome])
Diabetes	C1541814	Diabetes + eye manifestation (& [cataract] or [retinopathy])
Diabetes	C1542035	Diabetes mellitus: [juvenile type, with no mention of complication] or [insulin dependent]
Diabetes	C1561823	Diabetes mellitus type I, uncontrolled, with neurological manifestations
Diabetes	C1642836	Infection of foot associated with diabetes
Diabetes	C1719759	Renal disorder associated with type I diabetes mellitus
Diabetes	C1719760	Peripheral circulatory disorder associated with diabetes mellitus
Diabetes	C1719769	Exudative maculopathy associated with type I diabetes mellitus
Diabetes	C1719783	Persistent proteinuria associated with type I diabetes mellitus
Diabetes	C1719807	Diabetic cataract associated with type I diabetes mellitus
Diabetes	C1719887	Persistent proteinuria associated with type II diabetes mellitus
Diabetes	C1719929	Persistent microalbuminuria associated with type I diabetes mellitus
Diabetes	C1719939	Disorder associated with type 2 diabetes mellitus
Diabetes	C1719950	Diabetic retinopathy associated with type II diabetes mellitus
Diabetes	C1719988	Neurologic disorder associated with type II diabetes mellitus
Diabetes	C1720029	Coma associated with diabetes mellitus
Diabetes	C1720042	Neurological disorder associated with malnutrition-related diabetes mellitus
Diabetes	C1720056	Peripheral circulatory disorder associated with type I diabetes mellitus
Diabetes	C1720070	Gestational diabetes mellitus, class C
Diabetes	C1720078	Neurologic disorder associated with diabetes mellitus
Diabetes	C1720102	Persistent microalbuminuria associated with type II diabetes mellitus
Diabetes	C1720165	Peripheral circulatory disorder associated with type II diabetes mellitus
Diabetes	C1720171	Diabetic cataract associated with type II diabetes mellitus
Diabetes	C1720194	Neurological disorder associated with type I diabetes mellitus
Diabetes	C1720223	Diabetic retinopathy associated with type I diabetes mellitus
Diabetes	C1720297	Disorder associated with type I diabetes mellitus
Diabetes	C1720380	Polyneuropathy associated with type I diabetes mellitus
Diabetes	C1720409	Mononeuropathy associated with type I diabetes mellitus
Diabetes	C1720457	Renal disorder associated with type II diabetes mellitus
Diabetes	C1720489	Coma associated with malnutrition-related diabetes mellitus
Diabetes	C1720557	Polyneuropathy associated with type II diabetes mellitus
Diabetes	C1720626	Exudative maculopathy associated with type II diabetes mellitus
Diabetes	C1720648	Mononeuropathy associated with type II diabetes mellitus
Diabetes	C1720717	Diabetic oculopathy associated with type I diabetes mellitus
Diabetes	C1827179	Diabetic gastroparesis associated with type 2 diabetes mellitus
Diabetes	C1827566	Diabetic gastroparesis associated with type 1 diabetes mellitus
Diabetes	C1827612	Diabetic autonomic neuropathy associated with type 2 diabetes mellitus
Diabetes	C1832387	DIABETES MELLITUS, NONINSULIN-DEPENDENT, 2 (disorder)
Diabetes	C1832392	DIABETES MELLITUS, INSULIN-DEPENDENT, 12
Diabetes	C1832474	DIABETES MELLITUS, INSULIN-DEPENDENT, 13
Diabetes	C1832544	DIABETES MELLITUS, NONINSULIN-DEPENDENT, 1 (disorder)
Diabetes	C1832605	DIABETES MELLITUS, INSULIN-DEPENDENT, 11

Diabetes	C1833218	DIABETES MELLITUS, INSULIN-DEPENDENT, 8
Diabetes	C1838259	DIABETES MELLITUS, INSULIN-DEPENDENT, 7
Diabetes	C1838260	DIABETES MELLITUS, INSULIN-DEPENDENT, 5
Diabetes	C1838261	DIABETES MELLITUS, INSULIN-DEPENDENT, 4
Diabetes	C1838262	DIABETES MELLITUS, INSULIN-DEPENDENT, 3
Diabetes	C1842642	DIABETES MELLITUS, NONINSULIN-DEPENDENT, 4
Diabetes	C1852092	DIABETES MELLITUS, INSULIN-DEPENDENT, 2
Diabetes	C1854125	DIABETES MELLITUS, INSULIN-DEPENDENT, 18
Diabetes	C1857808	DIABETES MELLITUS, INSULIN-DEPENDENT, 19 (disorder)
Diabetes	C1863594	DIABETES MELLITUS, NONINSULIN-DEPENDENT, 3
Diabetes	C1864068	DIABETES MELLITUS, INSULIN-DEPENDENT, 17
Diabetes	C1866040	DIABETES MELLITUS, INSULIN-DEPENDENT, 10
Diabetes	C1866041	DIABETES MELLITUS, INSULIN-DEPENDENT, 6
Diabetes	C1866519	DIABETES MELLITUS, INSULIN-DEPENDENT, 15
Diabetes	C1960041	Small vessel disease due to type 1 diabetes mellitus
Diabetes	C1960042	Small vessel disease due to type 2 diabetes mellitus
Diabetes	C1960216	Amyotrophy due to type 1 diabetes mellitus
Diabetes	C1960217	Amyotrophy due to type 2 diabetes mellitus
Diabetes	C1960272	Latent autoimmune diabetes mellitus in adult
Diabetes	C1960678	Diabetic autonomic neuropathy associated with type 1 diabetes mellitus
Diabetes	C1997115	Hyperosmolality due to uncontrolled type 1 diabetes mellitus
Diabetes	C1997651	Erectile dysfunction associated with type 2 diabetes mellitus
Diabetes	C2004233	Diabetes mellitus: [with renal manifestation] or [nephropathy]
Diabetes	C2004234	Diabetes mellitus with: [gangrene] or [peripheral circulatory disorder]
Diabetes	C2118433	diabetes mellitus with postsurgical hypoinsulinemia
Diabetes	C2119283	uncontrolled type II diabetes mellitus with peripheral neuropathy (diagnosis)
Diabetes	C2119284	uncontrolled type I diabetes mellitus with peripheral neuropathy (diagnosis)
Diabetes	C2242974	Diabetes mellitus with neurological manifestation: (& [amyotrophy] or [neuropathy] or [polyneuropathy])
Diabetes	C2349362	Secondary diabetes mellitus, uncontrolled
Diabetes	C2349363	Secondary diabetes mellitus, without mention of complication
Diabetes	C2349364	Secondary diabetes mellitus without mention of complication or manifestation classifiable to 249.1-249.9
Diabetes	C2349365	Secondary diabetes mellitus with ketoacidosis, not stated as uncontrolled, or unspecified
Diabetes	C2349366	Secondary diabetes mellitus with ketoacidosis, uncontrolled
Diabetes	C2349367	secondary diabetes mellitus with ketoacidosis
Diabetes	C2349368	Secondary diabetes mellitus with diabetic acidosis without mention of coma
Diabetes	C2349369	Secondary diabetes mellitus with diabetic ketosis without mention of coma
Diabetes	C2349370	Secondary diabetes mellitus with hyperosmolarity, not stated as uncontrolled, or unspecified
Diabetes	C2349371	Secondary diabetes mellitus with hyperosmolarity, uncontrolled
Diabetes	C2349372	secondary diabetes mellitus with hyperosmolarity
Diabetes	C2349380	Secondary diabetes mellitus with renal manifestations, not stated as uncontrolled, or unspecified
Diabetes	C2349381	Secondary diabetes mellitus with renal manifestations, uncontrolled
Diabetes	C2349382	secondary diabetes mellitus with renal manifestations
Diabetes	C2349383	Secondary diabetes mellitus with ophthalmic manifestations, not stated as uncontrolled, or unspecified
Diabetes	C2349384	Secondary diabetes mellitus with ophthalmic manifestations, uncontrolled
Diabetes	C2349385	secondary diabetes mellitus with ophthalmic manifestations
Diabetes	C2349386	Secondary diabetes mellitus with neurological manifestations, not stated as uncontrolled, or unspecified
Diabetes	C2349387	Secondary diabetes mellitus with neurological manifestations, uncontrolled
Diabetes	C2349388	secondary diabetes mellitus with neurological manifestations
Diabetes	C2349389	Secondary diabetes mellitus with peripheral circulatory disorders, not stated as uncontrolled, or unspecified
Diabetes	C2349390	Secondary diabetes mellitus with peripheral circulatory disorders, uncontrolled
Diabetes	C2349391	secondary diabetes mellitus with peripheral circulatory disorders
Diabetes	C2349392	Secondary diabetes mellitus with other specified manifestations, not stated as uncontrolled, or unspecified
Diabetes	C2349393	Secondary diabetes mellitus with other specified manifestations, uncontrolled
Diabetes	C2349394	Secondary diabetes mellitus with other specified manifestations
Diabetes	C2349395	Secondary diabetic hypoglycemia in diabetes mellitus
Diabetes	C2349397	Secondary diabetes mellitus with unspecified complication, not stated as uncontrolled, or unspecified
Diabetes	C2349398	Secondary diabetes mellitus with unspecified complication, uncontrolled
Diabetes	C2349399	Secondary diabetes mellitus with unspecified complication
Diabetes	C2362516	Sugar Diabetes
Diabetes	C2675472	DIABETES MELLITUS, INSULIN-DEPENDENT, 23
Diabetes	C2675864	DIABETES MELLITUS, INSULIN-DEPENDENT, 22 (disorder)
Diabetes	C2675865	DIABETES MELLITUS, INSULIN-DEPENDENT, 21 (disorder)

Diabetes	C2675866	DIABETES MELLITUS, INSULIN-DEPENDENT, 20 (disorder)
Diabetes	C2676834	MICROVASCULAR COMPLICATIONS OF DIABETES, PROTECTION FROM
Diabetes	C2711084	Hyperglycemic crisis in diabetes mellitus
Diabetes	C2711205	Multiple complications due to diabetes mellitus
Diabetes	C2919802	Brittle type II diabetes mellitus
Emphysema	C0013994	Interstitial emphysema of lung
Emphysema	C0034067	Pulmonary Emphysema
Emphysema	C0155872	Catarrhal bronchitis
Emphysema	C0155874	Emphysematous bronchitis
Emphysema	C0155875	Obstructive chronic bronchitis, without exacerbation
Emphysema	C0155898	Chronic respiratory condition due to fumes AND/OR vapors
Emphysema	C0155918	Compensatory emphysema
Emphysema	C0221227	Centriacinar Emphysema
Emphysema	C0241876	Obstructive emphysema
Emphysema	C0241877	EMPHYSEMA, PULMONARY BULLOUS
Emphysema	C0264393	Panacinar Emphysema
Emphysema	C0264394	Paraseptal emphysema
Emphysema	C0264396	Emphysematous bleb of lung
Emphysema	C0264397	Ruptured emphysematous bleb of lung
Emphysema	C0264398	Giant bullous emphysema
Emphysema	C0264459	Chronic diffuse emphysema due to inhalation of chemical fumes AND/OR vapors
Emphysema	C0340049	Atrophic (senile) emphysema
Emphysema	C0340051	Toxic emphysema
Emphysema	C0340052	Chronic emphysema due to chemical fumes
Emphysema	C0340054	Bullous emphysema with collapse
Emphysema	C0340055	Segmental bullous emphysema
Emphysema	C0340056	Zonal bullous emphysema
Emphysema	C0340057	Scar emphysema
Emphysema	C0853845	Pulmonary interstitial emphysema syndrome
Emphysema	C0865799	Lung or pulmonary emphysema , NOS
Emphysema	C0865808	Emphysema due to inhalation of chemical fumes and vapors
Emphysema	C0865809	Diffuse emphysema due to inhalation of chemical fumes and vapors
Emphysema	C0865810	Chronic emphysema due to inhalation of chemical fumes and vapors
Emphysema	C1261120	Chronic bullous emphysema
Emphysema	C1265792	Chronic emphysema
Emphysema	C1717804	Emphysema or COPD
Emphysema	C1851718	EMPHYSEMA, HEREDITARY PULMONARY
Emphysema	C2350878	Focal Emphysema
Heart failure	C0007222	Cardiovascular Diseases
Heart failure	C0018799	Heart Diseases
Heart failure	C0018802	Congestive heart failure
Heart failure	C0023212	Left-Sided Heart Failure
Heart failure	C0152105	Hypertensive heart disease
Heart failure	C0155586	Malignant hypertensive heart disease with congestive heart failure
Heart failure	C0155589	Benign hypertensive heart disease with congestive cardiac failure
Heart failure	C0155609	benign hypertensive heart and renal disease with congestive heart failure
Heart failure	C0155611	benign hypertensive heart and renal disease with congestive heart failure and renal failure
Heart failure	C0221045	High output heart failure
Heart failure	C0235525	DECOMPENSATION MYOCARDIAL
Heart failure	C0235527	Heart Failure, Right-Sided
Heart failure	C0264546	Pleural effusion due to congestive heart failure
Heart failure	C0264650	Hypertensive heart disease with congestive heart failure
Heart failure	C0264652	Hypertensive heart failure
Heart failure	C0264714	Acute heart failure
Heart failure	C0264715	Acute right heart failure
Heart failure	C0264716	Chronic heart failure
Heart failure	C0264717	Chronic right-sided heart failure
Heart failure	C0264719	Acute congestive heart failure
Heart failure	C0264720	Acute right-sided congestive heart failure
Heart failure	C0264721	Acute left-sided congestive heart failure
Heart failure	C0264722	Chronic congestive heart failure
Heart failure	C0264723	Right heart failure secondary to left heart failure

Heart failure	C0264724	Chronic right-sided congestive heart failure
Heart failure	C0264725	Acute left-sided heart failure
Heart failure	C0264726	Chronic left-sided heart failure
Heart failure	C0264727	Chronic left-sided congestive heart failure
Heart failure	C0264728	Low output heart failure
Heart failure	C0340510	Heart failure as a complication of care
Heart failure	C0392173	Heart insuffiency
Heart failure	C0494575	Hypertensive heart and renal disease with (congestive) heart failure
Heart failure	C0494576	Hypertensive heart and renal disease with both (congestive) heart failure and renal failure
Heart failure	C0518970	CARDIAC FAILURE RIGHT CONGESTIVE
Heart failure	C0685095	Biventricular congestive heart failure
Heart failure	C0742747	High-output congestive heart failure
Heart failure	C0742749	left sided congestive heart failure
Heart failure	C0742758	congestive heart failure symptom
Heart failure	C0810005	Congestive heart failure; nonhypertensive
Heart failure	C0859775	Hypertensive heart and renal disease, unspec, w/o ment of congestive heart failure or renal failure
Heart failure	C0865679	Acute pulmonary edema with heart disease NOS or heart failure
Heart failure	C1135191	Heart Failure, Systolic
Heart failure	C1135194	Chronic systolic heart failure
Heart failure	C1135196	Heart Failure, Diastolic
Heart failure	C1135329	Malignant hypertensive heart disease with heart failure
Heart failure	C1135331	Benign hypertensive heart disease with heart failure
Heart failure	C1135333	Unspecified hypertensive heart disease with heart failure
Heart failure	C1281998	Refractory heart failure
Heart failure	C1306063	Acute left ventricular failure
Heart failure	C1541842	Heart failure: [right] or [congestive]
Heart failure	C1541843	(Congestive cardiac failure) or (dropsy) or (cardiac insufficiency) or (right heart failure) or (right ventricular failure)
Heart failure	C1827266	Decompensated chronic heart failure
Heart failure	C1956414	Cardiac asthma
Heart failure	C1960707	Congestive heart failure due to valvular disease
Heart failure	C1961050	Congestive heart failure due to left ventricular systolic dysfunction
Heart failure	C1961112	Heart Decompensation
Heart failure	C2074673	chronic diastolic congestive heart failure
Heart failure	C2215111	acute diastolic congestive heart failure
Heart failure	C2215174	acute on chronic diastolic congestive heart failure
Heart failure	C2215175	acute on chronic systolic congestive heart failure
Heart failure	C2215291	acute systolic congestive heart failure
Heart failure	C2711480	Chronic diastolic heart failure
Heart failure	C2732748	Acute systolic heart failure
Heart failure	C2732749	Acute on chronic diastolic heart failure
Heart failure	C2732951	Acute diastolic heart failure
Heart failure	C2733492	Acute on chronic systolic heart failure
Heart failure	C2882273	Combined systolic and diastolic heart failure, unspecified
Heart failure	C2882274	Combined systolic and diastolic heart failure, acute
Heart failure	C2882275	Combined systolic and diastolic heart failure, chronic
Heart failure	C2882276	Combined systolic and diastolic heart failure, acute on chronic
Heart failure	C2939447	Right ventricular failure
Heart failure	C2960127	Heart failure with normal ejection fraction
Heart failure	No CUI for this Mesh id	mesh:D017095
Ischemic heart disease	C0002962	Angina Pectoris
Ischemic heart disease	C0002963	Angina Pectoris, Variant
Ischemic heart disease	C0010054	Coronary Arteriosclerosis
Ischemic heart disease	C0010068	Coronary heart disease
Ischemic heart disease	C0010072	Coronary Thrombosis
Ischemic heart disease	C0010073	Coronary Artery Vasospasm
Ischemic heart disease	C0018813	Myocardial rupture
Ischemic heart disease	C0018814	Heart Rupture, Post-Infarction
Ischemic heart disease	C0027051	Myocardial Infarction
Ischemic heart disease	C0151744	Myocardial Ischemia
Ischemic heart disease	C0151814	Coronary Occlusion
Ischemic heart disease	C0152107	Postmyocardial infarction syndrome
Ischemic heart disease	C0155626	Acute myocardial infarction

Ischemic heart disease	C0155627	Acute myocardial infarction of anterolateral wall
Ischemic heart disease	C0155628	Acute myocardial infarction, of anterolateral wall, episode of care unspecified
Ischemic heart disease	C0155629	Acute myocardial infarction, of anterolateral wall, initial episode of care
Ischemic heart disease	C0155630	Acute myocardial infarction, of anterolateral wall, subsequent episode of care
Ischemic heart disease	C0155631	Acute myocardial infarction, of other anterior wall
Ischemic heart disease	C0155632	Acute myocardial infarction, of other anterior wall, episode of care unspecified
Ischemic heart disease	C0155633	Acute myocardial infarction, of other anterior wall, initial episode of care
Ischemic heart disease	C0155634	Acute myocardial infarction, of other anterior wall, subsequent episode of care
Ischemic heart disease	C0155636	Acute myocardial infarction, of inferolateral wall, episode of care unspecified
Ischemic heart disease	C0155637	Acute myocardial infarction, of inferolateral wall, initial episode of care
Ischemic heart disease	C0155638	Acute myocardial infarction, of inferolateral wall, subsequent episode of care
Ischemic heart disease	C0155640	Acute myocardial infarction, of inferoposterior wall, episode of care unspecified
Ischemic heart disease	C0155641	Acute myocardial infarction, of inferoposterior wall, initial episode of care
Ischemic heart disease	C0155642	Acute myocardial infarction, of inferoposterior wall, subsequent episode of care
Ischemic heart disease	C0155643	Acute myocardial infarction, of other inferior wall
Ischemic heart disease	C0155644	Acute myocardial infarction, of other inferior wall, episode of care unspecified
Ischemic heart disease	C0155645	Acute myocardial infarction, of other inferior wall, initial episode of care
Ischemic heart disease	C0155646	Acute myocardial infarction, of other inferior wall, subsequent episode of care
Ischemic heart disease	C0155647	Acute myocardial infarction, of other lateral wall
Ischemic heart disease	C0155648	Acute myocardial infarction, of other lateral wall, episode of care unspecified
Ischemic heart disease	C0155649	Acute myocardial infarction, of other lateral wall, initial episode of care
Ischemic heart disease	C0155650	Acute myocardial infarction, of other lateral wall, subsequent episode of care
Ischemic heart disease	C0155652	Acute myocardial infarction, true posterior wall infarction, episode of care unspecified
Ischemic heart disease	C0155653	Acute myocardial infarction, true posterior wall infarction, initial episode of care
Ischemic heart disease	C0155654	Acute myocardial infarction, true posterior wall infarction, subsequent episode of care
Ischemic heart disease	C0155655	Acute myocardial infarction, subendocardial infarction
Ischemic heart disease	C0155657	Acute myocardial infarction, subendocardial infarction, initial episode of care
Ischemic heart disease	C0155658	Acute myocardial infarction, subendocardial infarction, subsequent episode of care
Ischemic heart disease	C0155659	Acute myocardial infarction, of other specified sites
Ischemic heart disease	C0155660	Acute myocardial infarction, of other specified sites, episode of care unspecified
Ischemic heart disease	C0155661	Acute myocardial infarction, of other specified sites, initial episode of care
Ischemic heart disease	C0155662	Acute myocardial infarction, of other specified sites, subsequent episode of care
Ischemic heart disease	C0155664	Acute myocardial infarction, unspecified site, initial episode of care
Ischemic heart disease	C0155665	Acute myocardial infarction, unspecified site, subsequent episode of care
Ischemic heart disease	C0155668	Old myocardial infarction
Ischemic heart disease	C0155669	Other/chronic ischemic heart disease
Ischemic heart disease	C0155670	Other specified forms of chronic ischemic heart disease
Ischemic heart disease	C0206064	Microvascular Angina
Ischemic heart disease	C0235462	ANGINA ATTACK
Ischemic heart disease	C0238414	Mitral papillary muscle rupture
Ischemic heart disease	C0262563	MYOCARDIAL INFARCTION (ANTERIOR WALL) (SUBENDOCARDIAL)
Ischemic heart disease	C0262564	Anterolateral Myocardial Infarction
Ischemic heart disease	C0262565	Anteroseptal Myocardial Infarction
Ischemic heart disease	C0262567	MYOCARDIAL INFARCTION (INFERIOR WALL) (TRUE POSTERIOR)
Ischemic heart disease	C0262568	Subendocardial myocardial infarction
Ischemic heart disease	C0264674	Acute infarction of papillary muscle
Ischemic heart disease	C0264693	Acute coronary insufficiency
Ischemic heart disease	C0264694	Chronic myocardial ischemia
Ischemic heart disease	C0264695	Subendocardial ischemia
Ischemic heart disease	C0264698	Acute anteroapical myocardial infarction
Ischemic heart disease	C0264699	Acute anteroseptal myocardial infarction
Ischemic heart disease	C0264700	Acute Inferior Myocardial Infarction
Ischemic heart disease	C0264702	Acute myocardial infarction of apical-lateral wall
Ischemic heart disease	C0264703	Acute myocardial infarction of basal-lateral wall
Ischemic heart disease	C0264704	Acute myocardial infarction of high lateral wall
Ischemic heart disease	C0264705	Acute myocardial infarction of posterolateral wall
Ischemic heart disease	C0264706	True posterior myocardial infarction
Ischemic heart disease	C0264707	Acute myocardial infarction of posterobasal wall
Ischemic heart disease	C0264708	Acute myocardial infarction of atrium
Ischemic heart disease	C0281915	Lateral Wall Myocardial Infarction
Ischemic heart disease	C0340283	Other acute and subacute forms of ischemic heart disease
Ischemic heart disease	C0340286	Other specified chronic ischemic heart disease NOS

Ischemic heart disease	C0340287	Other specified ischemic heart disease
Ischemic heart disease	C0340292	Other specified anterior myocardial infarction
Ischemic heart disease	C0340293	Anterior myocardial infarction
Ischemic heart disease	C0340294	Other acute myocardial infarction NOS
Ischemic heart disease	C0340295	Acute anteroapical infarction
Ischemic heart disease	C0340297	Acute myocardial infarction of septum
Ischemic heart disease	C0340304	Acute myocardial infarction of inferoposterior wall
Ischemic heart disease	C0340305	Inferior Wall Myocardial Infarction
Ischemic heart disease	C0340308	Acute myocardial infarction of inferolateral wall
Ischemic heart disease	C0340311	Acute myocardial infarction of lateral wall
Ischemic heart disease	C0340312	Lateral myocardial infarction
Ischemic heart disease	C0340315	Acute widespread myocardial infarction
Ischemic heart disease	C0340318	Acute posterior myocardial infarction
Ischemic heart disease	C0340319	Posterior myocardial infarction
Ischemic heart disease	C0340320	Old anterior myocardial infarction
Ischemic heart disease	C0340321	Old inferior myocardial infarction
Ischemic heart disease	C0340322	Old lateral myocardial infarction
Ischemic heart disease	C0340323	Old posterior myocardial infarction
Ischemic heart disease	C0340324	Silent myocardial infarction
Ischemic heart disease	C0340332	Cardiac rupture after acute myocardial infarction
Ischemic heart disease	C0340366	Mitral chordae rupture
Ischemic heart disease	C0348588	Other and unspecified angina pectoris
Ischemic heart disease	C0348590	Other forms of acute ischemic heart disease
Ischemic heart disease	C0348591	Acute transmural myocardial infarction of unspecified site
Ischemic heart disease	C0348592	Subsequent myocardial infarction of other sites
Ischemic heart disease	C0348593	Subsequent myocardial infarction
Ischemic heart disease	C0348862	Subsequent myocardial infarction of anterior wall
Ischemic heart disease	C0348863	Subsequent myocardial infarction of inferior wall
Ischemic heart disease	C0494580	Acute myocardial infarction, subendocardial infarction, episode of care unspecified
Ischemic heart disease	C0519049	PERICARDITIS ACUTE NONSUPPURATIVE
Ischemic heart disease	C0520544	Acute myocardial infarction with rupture of ventricle
Ischemic heart disease	C0541777	ANGINA AT REST PRINZMETAL'S
Ischemic heart disease	C0542052	Coronary insufficiency
Ischemic heart disease	C0542060	ISCHEMIA CORONARY ARTERY ORIGIN
Ischemic heart disease	C0542269	Non-Q wave myocardial infarction
Ischemic heart disease	C0565816	Acute/subacute ischemic heart disease NOS
Ischemic heart disease	C0577698	Exercise-induced angina
Ischemic heart disease	C0582803	Acute Q wave myocardial infarction
Ischemic heart disease	C0589363	Postoperative transmural myocardial infarction of anterior wall
Ischemic heart disease	C0589364	Postoperative transmural myocardial infarction of inferior wall
Ischemic heart disease	C0589365	Postoperative transmural myocardial infarction of other sites
Ischemic heart disease	C0589366	Postoperative transmural myocardial infarction of unspecified site
Ischemic heart disease	C0589367	Postoperative subendocardial myocardial infarction
Ischemic heart disease	C0589368	Postoperative myocardial infarction
Ischemic heart disease	C0746727	Septal myocardial infarction
Ischemic heart disease	C0865592	Acute myocardial infarction, anteroapical with contiguous portion of intraventricular septum
Ischemic heart disease	C0865593	Acute myocardial infarction, anteroseptal with contiguous portion of intraventricular septum
Ischemic heart disease	C0865605	Acute coronary embolism without or not resulting in myocardial infarction
Ischemic heart disease	C0865607	Acute coronary thrombosis without or not resulting in myocardial infarction
Ischemic heart disease	C0948089	Acute Coronary Syndrome
Ischemic heart disease	C0949167	Acute coronary occlusion without myocardial infarction
Ischemic heart disease	C1112662	Posterolateral myocardial infarction
Ischemic heart disease	C1112770	Inferolateral myocardial infarction
Ischemic heart disease	C1142433	Posteroinferior myocardial infarction
Ischemic heart disease	C1168331	Apical myocardial infarction
Ischemic heart disease	C1273976	First myocardial infarction
Ischemic heart disease	C1276061	Acute non-ST segment elevation myocardial infarction
Ischemic heart disease	C1299620	Myocardial infarction with complication
Ischemic heart disease	C1303258	Acute ST segment elevation myocardial infarction
Ischemic heart disease	C1510446	Acute ischemic heart disease
Ischemic heart disease	C1534921	(Myocardial infarction (& [acute] or [silent] or [cardiac rupture following])) or (coronary thrombosis)
Ischemic heart disease	C1534922	Myocardial infarction (& [acute]) or coronary thrombosis

Ischemic heart disease	C1561914	ST elevation myocardial infarction (STEMI) of anterolateral wall
Ischemic heart disease	C1561915	ST elevation myocardial infarction (STEMI) of other anterior wall
Ischemic heart disease	C1561916	ST elevation myocardial infarction (STEMI) of inferolateral wall
Ischemic heart disease	C1561917	ST elevation myocardial infarction (STEMI) of inferoposterior wall
Ischemic heart disease	C1561918	ST elevation myocardial infarction (STEMI) of other inferior wall
Ischemic heart disease	C1561919	ST elevation myocardial infarction (STEMI) of other lateral wall
Ischemic heart disease	C1561920	ST elevation myocardial infarction (STEMI) of true posterior wall
Ischemic heart disease	C1561921	Non-ST elevation myocardial infarction (NSTEMI)
Ischemic heart disease	C1561922	ST elevation myocardial infarction (STEMI) of other specified sites
Ischemic heart disease	C1561923	ST elevation (STEMI) and non-ST elevation (NSTEMI) myocardial infarction
Ischemic heart disease	C1837871	MYOCARDIAL INFARCTION, SUSCEPTIBILITY TO, 2
Ischemic heart disease	C1956346	Coronary Artery Disease
Ischemic heart disease	C2003997	Ischemic heart disease (& [arteriosclerotic])
Ischemic heart disease	C2349195	Acute Anterior Wall Myocardial Infarction
Lung cancer	C0001418	Adenocarcinoma
Lung cancer	C0006264	Bronchial Neoplasms
Lung cancer	C0007120	Bronchioloalveolar Carcinoma
Lung cancer	C0007121	Bronchogenic Carcinoma
Lung cancer	C0007131	Carcinoma, Non-Small-Cell Lung
Lung cancer	C0007137	Squamous cell carcinoma
Lung cancer	C0024121	Lung Neoplasms
Lung cancer	C0149782	Squamous cell carcinoma of lung
Lung cancer	C0149925	Small cell carcinoma of lung
Lung cancer	C0152013	Adenocarcinoma of lung (disorder)
Lung cancer	C0153676	Secondary malignant neoplasm of lung
Lung cancer	C0154071	Carcinoma in situ of bronchus and lung
Lung cancer	C0238254	Metastatic Carcinoma to the Lung
Lung cancer	C0242379	Malignant neoplasm of lung
Lung cancer	C0278503	occult non-small cell lung cancer
Lung cancer	C0278504	Non-small cell lung cancer stage I
Lung cancer	C0278505	Non-small cell lung cancer stage II
Lung cancer	C0278506	Non-small cell lung cancer stage III
Lung cancer	C0278517	Non-small cell lung cancer recurrent
Lung cancer	C0278725	limited stage small cell lung cancer
Lung cancer	C0278726	extensive stage small cell lung cancer
Lung cancer	C0278727	recurrent small cell lung cancer
Lung cancer	C0278983	Non-small cell lung cancer stage IIIA
Lung cancer	C0278984	Non-small cell lung cancer stage IIIB
Lung cancer	C0278986	Non-small cell lung cancer stage 0
Lung cancer	C0278987	Non-small cell lung cancer metastatic
Lung cancer	C0279557	adenosquamous cell lung cancer
Lung cancer	C0279568	lymphocyte-like type small cell lung cancer
Lung cancer	C0279569	intermediate type small cell lung cancer
Lung cancer	C0279570	polygonal type small cell lung cancer
Lung cancer	C0279571	fusiform type small cell lung cancer
Lung cancer	C0279572	combined type small cell lung cancer
Lung cancer	C0279877	cellular diagnosis, non-small cell lung cancer
Lung cancer	C0279902	cellular diagnosis, small cell lung cancer
Lung cancer	C0280217	stage, non-small cell lung cancer
Lung cancer	C0280249	stage, small cell lung cancer
Lung cancer	C0345958	Large cell carcinoma of lung
Lung cancer	C0346961	Secondary malignant neoplasm of bronchus
Lung cancer	C0518964	BRONCHIAL ADENOCARCINOMA
Lung cancer	C0519064	PULMONARY MALIGNANT NEOPLASM SECONDARY HEMATOGENOUS TYPE
Lung cancer	C0684249	Carcinoma of lung
Lung cancer	C0685053	Carcinoma in situ of lung
Lung cancer	C0815311	Pulmonary Lunatism
Lung cancer	C0854978	Large cell lung cancer stage 0
Lung cancer	C0855002	Lung carcinoma cell type unspecified recurrent
Lung cancer	C0855005	Lung carcinoma cell type unspecified stage IV
Lung cancer	C0862802	Lung cancer recurrent
Lung cancer	C0862824	Lung cancer stage I

Lung cancer	C0862833	Lung cancer stage II
Lung cancer	C0862847	Lung cancer stage III
Lung cancer	C1314696	Squamous cell carcinoma of bronchus
Lung cancer	C1333125	combined small cell carcinoma of lung
Lung cancer	C1335098	Occult Non-Small Cell Lung Carcinoma
Lung cancer	C1336119	Stage IA Non-Small Cell Lung Carcinoma
Lung cancer	C1336124	Stage IA Small Cell Lung Carcinoma
Lung cancer	C1336139	Stage IB Non-Small Cell Lung Carcinoma
Lung cancer	C1336144	Stage IB Small Cell Lung Carcinoma
Lung cancer	C1336167	Stage IIA Non-Small Cell Lung Carcinoma
Lung cancer	C1336172	Stage IIA Small Cell Lung Carcinoma
Lung cancer	C1336190	Stage IIB Non-Small Cell Lung Carcinoma
Lung cancer	C1336195	Stage IIB Small Cell Lung Carcinoma
Lung cancer	C1336215	Stage IIIA Small Cell Lung Carcinoma
Lung cancer	C1336230	Stage IIIB Small Cell Lung Carcinoma
Lung cancer	C1336231	Stage IIIB Small Cell Lung Carcinoma with Pleural Effusion
Lung cancer	C1336232	Stage IIIB Small Cell Lung Carcinoma without Pleural Effusion
Lung cancer	C1336271	Stage III Small Cell Lung Carcinoma
Lung cancer	C1336318	Stage II Small Cell Lung Carcinoma
Lung cancer	C1336440	Stage IV Small Cell Lung Carcinoma
Lung cancer	C1336482	Stage I Small Cell Lung Carcinoma
Lung cancer	C1578781	Malignant neoplasm lung: [of bronchus or lung NOS] or [lung cancer]
Lung cancer	C1837089	LUNG CANCER SUSCEPTIBILITY 1
Lung cancer	C1960396	Epithelial growth factor receptor negative non-small cell lung cancer
Lung cancer	C1960925	Epithelial growth factor receptor positive non-small cell lung cancer
Lung cancer	C2239115	non-oat cell lung cancer, large cell
Lung cancer	C2675479	LUNG CANCER SUSCEPTIBILITY 4
Lung cancer	C2675497	LUNG CANCER SUSCEPTIBILITY 3
Lung cancer	C2677571	LUNG CANCER SUSCEPTIBILITY 2 (disorder)
Lung cancer	C2981344	Stage I Lung Cancer AJCC v6
Lung cancer	C2981348	Stage III Lung Cancer AJCC v6
Lung cancer	C2981352	Stage IV Lung Cancer AJCC v6
Lung cancer	C2982916	Stage II Lung Cancer AJCC v6
Metabolic syndrome	C0020459	Hyperinsulinism
Metabolic syndrome	C0948265	Metabolic syndrome
Metabolic syndrome	C1262289	dysmetabolic syndrome
Metabolic syndrome	C1970051	METABOLIC SYNDROME, PROTECTION AGAINST
Muscle weakness	C0026827	Muscle hypotonia
Muscle weakness	C0026846	Muscular Atrophy
Muscle weakness	C0026848	Myopathy
Muscle weakness	C0030552	Paresis
Muscle weakness	C0151786	Muscle Weakness
Muscle weakness	C0234143	Neurological muscle weakness
Muscle weakness	C0746674	Generalized muscle weakness
Muscle weakness	C1560114	Whole Body and Generalized Muscle Weakness Not Due to Neuropathy Adverse Event
Obesity	C0028754	Obesity
Obesity	C0028756	Obesity, Morbid
Obesity	C0031880	Obesity Hypoventilation Syndrome
Obesity	C0149974	PRIMARY OBESITY
Obesity	C0154269	Obesity and other hyperalimentation
Obesity	C0267989	Lifelong obesity
Obesity	C0267990	Adult-onset obesity
Obesity	C0267992	Obesity of endocrine origin
Obesity	C0311277	Obesity, Abdominal
Obesity	C0342940	Android obesity
Obesity	C0342942	Generalized obesity
Obesity	C0348480	Other obesity
Obesity	C0451819	Simple obesity
Obesity	C0497406	Overweight
Obesity	C0524620	Metabolic Syndrome X
Obesity	C0545039	Hyperinsulinar obesity
Obesity	C0545040	Hyperplasmic obesity

Obesity	C0545041	Hypoplasmic obesity
Obesity	C0694533	Moderate obesity
Obesity	C0857116	gross obesity
Obesity	C1257763	Overnutrition
Obesity	C1260894	Hypertrophic obesity
Obesity	C1281429	Exogenous obesity
Obesity	C1281440	Familial obesity
Obesity	C1285391	Obesity associated disorder
Obesity	C1285438	Obesity associated adipose tissue distribution pattern
Obesity	C1532480	Hyperplastic obesity
Obesity	C1532609	Obesity by adipocyte growth pattern
Obesity	C1532610	Obesity by age of onset
Obesity	C1532611	Obesity by contributing factors
Obesity	C1532612	Obesity by fat distribution pattern
Obesity	C1542422	(Hyperalimentation including obesity) or (adiposity)
Obesity	C1561826	Overweight and obesity
Obesity	C1561827	Overweight, obesity and other hyperalimentation
Obesity	C1854178	ABDOMINAL OBESITY-METABOLIC SYNDROME QUANTITATIVE TRAIT LOCUS 1
Obesity	C1971023	Hyperalimentation incl.obesity
Obesity	C2675358	BODY MASS INDEX QUANTITATIVE TRAIT LOCUS 7 (disorder)
Obesity	C2675659	BODY MASS INDEX QUANTITATIVE TRAIT LOCUS 10 (disorder)
Obesity	C2675904	WILMS TUMOR, ANIRIDIA, GENITOURINARY ANOMALIES, MENTAL RETARDATION, AND OBESITY SYNDROME
Obesity	C2676498	BODY MASS INDEX QUANTITATIVE TRAIT LOCUS 12 (disorder)
Obesity	C2676933	BODY MASS INDEX QUANTITATIVE TRAIT LOCUS 8 (disorder)
Obesity	C2677162	BODY MASS INDEX QUANTITATIVE TRAIT LOCUS 9 (disorder)
Obesity	C2678155	BODY MASS INDEX QUANTITATIVE TRAIT LOCUS 11 (disorder)
Obesity	C2930930	Abdominal obesity metabolic syndrome
Obesity	C2936179	Obesity, Visceral
Obesity	C2937223	Hyperplastic-hypertrophic obesity
Obesity	C2937224	Constitutional obesity
Osteoporosis	C0001787	Osteoporosis, Age-Related
Osteoporosis	C0029453	Osteopenia
Osteoporosis	C0029456	Osteoporosis
Osteoporosis	C0029458	Osteoporosis, Postmenopausal
Osteoporosis	C0029459	Osteoporosis, Senile
Osteoporosis	C0029694	Other osteoporosis
Osteoporosis	C0152256	Disuse osteoporosis
Osteoporosis	C0158447	Idiopathic osteoporosis
Osteoporosis	C0262586	OSTEOPOROSIS (OSTEOPENIA)
Osteoporosis	C0264115	Drug-induced osteoporosis
Osteoporosis	C0302887	TRANSIENT REGIONAL OSTEOPOROSIS
Osteoporosis	C0343264	Idiopathic generalized osteoporosis
Osteoporosis	C0410438	Primary osteoporosis
Osteoporosis	C0410440	Regional migrating osteoporosis
Osteoporosis	C0451868	Localized osteoporosis - Lequesne
Osteoporosis	C0451869	Osteoporosis in endocrine disorders
Osteoporosis	C0451881	Osteoporosis of disuse with pathological fracture
Osteoporosis	C0451883	Drug-induced osteoporosis with pathological fracture
Osteoporosis	C0451884	Idiopathic osteoporosis with pathological fracture
Osteoporosis	C0473769	Adult idiopathic generalized osteoporosis
Osteoporosis	C0473771	Secondary generalized osteoporosis
Osteoporosis	C0473772	Secondary localized osteoporosis
Osteoporosis	C0473773	Localized disuse osteoporosis
Osteoporosis	C0477673	Other osteoporosis with pathological fracture
Osteoporosis	C0477674	Osteoporosis in other diseases classified elsewhere
Osteoporosis	C0560285	Osteoporosis with pathological fracture of lumbar vertebrae
Osteoporosis	C0560287	Osteoporosis with pathological fracture of thoracic vertebrae
Osteoporosis	C0560288	Osteoporosis with pathological fracture of cervical vertebrae
Osteoporosis	C0581321	Vertebral osteoporosis
Osteoporosis	C0587229	Osteoporosis localized to spine
Osteoporosis	C0747079	osteoporosis risk
Osteoporosis	C1272167	Osteoporosis due to corticosteroids

Osteoporosis	C1277186	Fragility fracture due to unspecified osteoporosis
Osteoporosis	C1866079	BONE MINERAL DENSITY QUANTITATIVE TRAIT LOCUS 1
Osteoporosis	C2316071	Pathologic fracture of femur associated with osteoporosis
Osteoporosis	C2317122	Pathological fracture of neck of femur associated with osteoporosis
Osteoporosis	C2733085	Pathological fracture due to osteoporosis
Polycythemia	C0032461	Polycythemia
Polycythemia	C0152264	Familial erythrocytosis
Polycythemia	C0221276	Relative erythrocytosis
Polycythemia	C0272139	Erythrocytosis due to low atmospheric pressure
Polycythemia	C0272144	Erythrocytosis due to tissue hypoxemia
Polycythemia	C0391869	Polycythemia due to excess erythopoetin production
Polycythemia	C0472787	Polycythemia due to cyanotic respiratory disease
Polycythemia	C0472789	Secondary polycythemia without excess erythropoietin
Polycythemia	C0852343	Polycythaemia (excl rubra vera)
Polycythemia	C0856817	Physiological polycythemia
Polycythemia	C0856818	Polycythemia due to hypoxia
Pulmonary hypertension	C0004468	Ayerza Syndrome
Pulmonary hypertension	C0020542	Pulmonary Hypertension
Pulmonary hypertension	C0152171	Primary pulmonary hypertension
Pulmonary hypertension	C0264934	Episodic pulmonary hypertension
Pulmonary hypertension	C0264935	Progressive pulmonary hypertension
Pulmonary hypertension	C0264936	Secondary pulmonary hypertension
Pulmonary hypertension	C0340540	Solitary pulmonary hypertension
Pulmonary hypertension	C0340541	Small vessel pulmonary hypertension
Pulmonary hypertension	C0340542	Sporadic primary pulmonary hypertension
Pulmonary hypertension	C0340543	Familial primary pulmonary hypertension
Pulmonary hypertension	C0340544	Drug-induced pulmonary hypertension
Pulmonary hypertension	C0340545	Large vessel pulmonary hypertension
Pulmonary hypertension	C0340546	Thromboembolic pulmonary hypertension
Pulmonary hypertension	C0340547	Post-arteritic pulmonary hypertension
Pulmonary hypertension	C0340549	Pulmonary hypertension associated with chronic underventilation
Pulmonary hypertension	C0340551	Pulmonary hypertension secondary to raised pulmonary vascular resistance
Pulmonary hypertension	C0349518	Pulmonary hypertension with extreme obesity
Pulmonary hypertension	C0349520	Facultative pulmonary hypertension with shunt at atrial level
Pulmonary hypertension	C1135361	Persistent pulmonary hypertension
Pulmonary hypertension	C1135362	Primary pulmonary hypertension of newborn
Pulmonary hypertension	C1971646	Pulmonary hypertension (& [primary])
Pulmonary hypertension	C2363973	Chronic thromboembolic pulmonary hypertension
Sleep apnea	C0020524	Disorders of Excessive Somnolence
Sleep apnea	C0020530	Hypersomnia with sleep apnea
Sleep apnea	C0037315	Sleep Apnea Syndromes
Sleep apnea	C0159030	Insomnia with sleep apnea
Sleep apnea	C0338495	Sleep Apnea, Mixed Central and Obstructive
Sleep apnea	C0338496	Alveolar sleep apnea
Sleep apnea	C0520679	Sleep Apnea, Obstructive
Sleep apnea	C0520680	Sleep Apnea, Central
Sleep apnea	C0751761	Upper Airway Resistance Sleep Apnea Syndrome
Sleep apnea	C0751762	Central Sleep Apnea, Primary
Sleep apnea	C0751763	Central Sleep Apnea, Secondary
Sleep apnea	C1135365	sleep; apnea, newborn
Sleep apnea	C1534531	([D]Hypersomnia with sleep apnoea) or (sleep apnoea syndrome)
Sleep apnea	C1561813	Insomnia with sleep apnea, unspecified
Sleep apnea	C1561815	Hypersomnia with sleep apnea, unspecified
Sleep apnea	C1561861	Organic sleep apnea
Sleep apnea	C1561868	Central sleep apnea in conditions classified elsewhere
Sleep apnea	C1561869	Other organic sleep apnea
Sleep apnea	C1842025	GLAUCOMA AND SLEEP APNEA
Stroke	C0001365	Acute ill-defined cerebrovascular disease
Stroke	C0007787	Transient Ischemic Attack
Stroke	C0018991	Hemiplegia
Stroke	C0038454	Cerebrovascular accident
Stroke	C0079102	Cerebral Thrombosis

Stroke	C0149854	Cerebellar hemorrhage
Stroke	C0151699	Intracranial Hemorrhages
Stroke	C0238281	Middle Cerebral Artery Syndrome
Stroke	C0241832	Cerebrovascular Insufficiency
Stroke	C0242129	Thrombotic stroke
Stroke	C0262469	Embolic stroke
Stroke	C0265082	Bulbar hemorrhage
Stroke	C0265084	Cerebromeningeal hemorrhage
Stroke	C0265085	Cortical hemorrhage
Stroke	C0265088	Intrapontine hemorrhage
Stroke	C0265112	Nonparalytic stroke
Stroke	C0265113	Progressing stroke
Stroke	C0265114	Completed stroke
Stroke	C0271373	Claude Syndrome
Stroke	C0281994	Stroke with Hemiparesis
Stroke	C0333559	Infarction, Lacunar
Stroke	C0393951	CVA - cerebrovascular accident due to cerebral artery occlusion
Stroke	C0393954	Total anterior cerebral circulation infarction
Stroke	C0393955	Partial anterior cerebral circulation infarction
Stroke	C0393963	Stroke of uncertain pathology
Stroke	C0393964	Anterior circulation stroke of uncertain pathology
Stroke	C0393965	Posterior circulation stroke of uncertain pathology
Stroke	C0393971	Spinal cord stroke
Stroke	C0451671	Brainstem stroke syndrome
Stroke	C0451672	Cerebellar stroke syndrome
Stroke	C0451680	Anterior Cerebral Artery Syndrome
Stroke	C0451681	Posterior Cerebral Artery Syndrome
Stroke	C0460035	Extension of cerebrovascular accident
Stroke	C0518974	CEREBRAL ARTERY THROMBOSIS OR DISSECTION WITH ENCEPHALOMALACIA
Stroke	C0521542	Brain Stem Infarctions
Stroke	C0542007	cerebral hematoma
Stroke	C0553692	Brain hemorrhage
Stroke	C0554399	Stroke in the puerperium
Stroke	C0596298	Cerebrovascular Occlusion
Stroke	C0740392	Infarction, Middle Cerebral Artery
Stroke	C0751843	Infarction, Anterior Cerebral Artery
Stroke	C0751956	Acute Cerebrovascular Accidents
Stroke	C0752132	Infarction, Posterior Cerebral Artery
Stroke	C0917798	Cerebral Ischemia
Stroke	C0948008	Ischemic stroke
Stroke	C1263853	Paralytic stroke
Stroke	C1298680	Occlusive stroke
Stroke	C1456577	Mini-Stroke
Stroke	C1531624	Cardioembolic stroke
Stroke	C1540598	(Cerebral infarct) or (cerebrovascular accident) or (undefined stroke/CVA) or (stroke NOS)
Stroke	C1579872	Cerebral arterial occlusion(&[cerebrovascular accident due to] or [stroke due to]) or (cerebral infarction)
Stroke	C1955572	Stroke NOS without residual deficits
Stroke	C1960869	Vertigo as late effect of stroke
Stroke	C2703075	stroke due to basilar artery occlusion
Stroke	C2931608	Anterior spinal artery stroke
Stroke	C2937358	Cerebral Hemorrhage
Stroke	C3178801	Stroke, Lacunar
